# Natural transaminase fusions for biocatalysis†

**DOI:** 10.1039/d3ra07081f

**Published:** 2024-01-31

**Authors:** Luba Prout, Helen C. Hailes, John M. Ward

**Affiliations:** a Department of Biochemical Engineering, University College London London WC1E 6BT UK j.ward@ucl.ac.uk; b Department of Chemistry, University College London 20 Gordon Street London WC1H 0AJ UK h.c.hailes@ucl.ac.uk

## Abstract

Biocatalytic approaches are used widely for the synthesis of amines from abundant or low cost starting materials. This is a fast-developing field where novel enzymes and enzyme combinations emerge quickly to enable the production of new and complex compounds. Natural multifunctional enzymes represent a part of multi-step biosynthetic pathways that ensure a one-way flux of reactants. *In vivo*, they confer a selective advantage *via* increased reaction rates and chemical stability or prevention of toxicity from reactive intermediates. Here we report the identification and analysis of a natural transaminase fusion, PP_2782, from *Pseudomonas putida* KT2440, as well as three of its thermophilic homologs from *Thermaerobacter marianensis*, *Thermaerobacter subterraneus*, and *Thermincola ferriacetica*. Both the fusions and their truncated transaminase-only derivatives showed good activity with unsubstituted aliphatic and aromatic aldehydes and amines, as well as with a range of α-keto acids, and l-alanine, l-glutamate, and l-glutamine. Through structural similarity, the fused domain was recognised as the acyl-[acyl-carrier-protein] reductase that affects reductive chain release. These natural transaminase fusions could have a great potential for industrial applications.

## Abstract 2

This work describes novel transaminase fusions, with potential for converting fatty acids into amines and more complex natural products.

## Graphical abstract

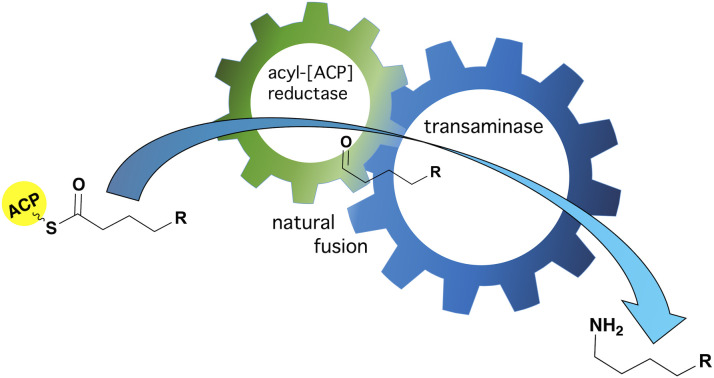

## Introduction

Manufacturing amine-containing pharmaceuticals and industrially important fine chemicals is essential for the production of a range of bioactive compounds. To circumvent challenges associated with chemical methods used for their production, enzymatic routes are being increasingly employed and developed as an alternative.^1–8^ A large number of natural biological catalysts representing a wide range of functionalities have been discovered and used for that purpose. Moreover, the acceleration of biocatalytic approaches has led to the development of multi-step enzyme cascades and *de novo* pathway engineering.^9–15^ These provide the means for generating complex compounds from abundant or cheap simple starting materials and are of increasing interest. However, in nature, enzymes have evolved to function together with other enzymes in specific environments, meaning that their isolation and use in non-native environments may reduce their efficiency. Typical issues include low yields due to unfavourable reaction equilibria, loss of intermediates to other cellular enzymes, toxicities, or substrate, product, or intermediate efflux in *in vivo* systems. The presence of multifunctional enzymes, which enable the metabolic channelling and sequestration of intermediates, suggests there is an evolutionary advantage to these systems.^16,17^ Such enzymes are often found within gene clusters or operons that are responsible for the synthesis of complex molecules, such as antibiotics or secondary metabolites.^18,19^ These multifunctional catalysts are often very efficient, having had the opportunity to evolve and improve over time.

**Fig. 1:**
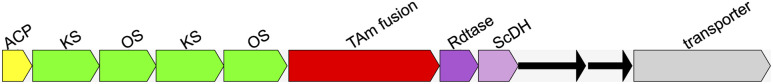
*P. putida* KT2440 PP_2777-PP_2787 gene cluster. Spanning over 14.7 kb, it is one of the two ‘atypical’ regions in the KT2440 genome thought to be associated with secondary metabolite production.^20^ ACP: acyl carrier protein; KS: β-ketoacyl synthase; OS: 3-oxoacyl-(ACP) synthase; TAm fusion: PLP-dependent TAm fusion; Rdtase: 3-oxoacyl-ACP reductase; ScDH: short-chain dehydrogenase. Thin black arrows represent proteins of unknown function.

Following our interest in transaminases (TAms), in preliminary studies, an open reading frame in the *Pseudomonas putida* KT2440 genome, specifically at locus PP_2782 (NCBI GenBank ref.: AAN68390.1, UniProt: Q88J67_PSEPK), was identified as encoding a TAm-like enzyme. The gene was unusually long compared to other known TAms and was found to contain a NAD(P)-binding region, suggesting a TAm-fused enzyme. Importantly, this fusion, which was termed *Pp*KTFusion, was found to also be located within a type II FAS/PKS gene cluster proposed to be involved in the biosynthesis of a secondary metabolite,^20^ (Fig. 1). Although previous studies could not determine the activity of the KT2440 PP_2777-PP_2787 loci,^20–22^ analogous gene clusters were identified in *Pseudomonas fluorescens* MC07,^23^ a forest soil metagenome DNA fragment,^22^*Pseudomonas* sp. SWI36 ^24^ and *Pseudomonas koreensis*.^25^ In those studies, the clusters were shown to play a pivotal role in the hosts' ability to exhibit antagonistic activity against either fungi^23^ or other bacteria.^24,25^

**Fig. 2:**
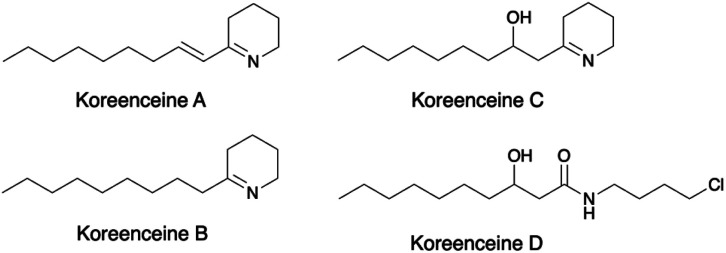
Tetrahydropyridine alkaloids isolated from *Pseudomonas koreensis*.^25^

Based on this biological activity, a new family of bacterial tetrahydropyridine alkaloids, koreenceines A to D (Fig. 2), was isolated from *P*. *koreensis*.^25^ Importantly, a transposon insertion in the PP_2782 homolog genes resulted in the complete loss of the organisms' antagonistic activity.^24,25^

Five- and six-membered nitrogen-containing heterocyclic compounds are common in nature, with many exhibiting important pharmacological properties. In recent years, increasing numbers of polyketide-derived piperidine alkaloids have been discovered in microorganisms, particularly in actinomycetes.^26,27^ Bacteria have been shown to utilise them for growth, colony differentiation, defence, pathogenicity, and quorum sensing.^26^ Many of these compounds also have antagonistic biological activities.^28^ Generally, in thiotemplate-based assembly lines, TAms are rare but may be present either as integrated domains within large multi-modular enzymes or as independent tailoring catalysts.^29–31^ Until recently, they were only known to occur in pathways associated with microcystins, iturins, and prodigiosins.^29^ However, a more recent analysis found that reductive chain release that is followed by a reductive transamination is a conserved mechanism in actinobacterial polyketide alkaloid biosynthesis.^31,32^ Gene clusters responsible for the synthesis of these alkaloids in actinomycetes comprise modular type I PKS and several core independent tailoring enzymes, including oxidoreductases, cyclases and a TAm.^31^ So far, the TAms identified were monofunctional enzymes that perform the conversion of a polyketide aldehyde to an amine.^28,32^

Generally, very few natural bifunctional TAm enzymes have been characterised and no thermophilic TAm fusions have previously been reported.^5,33,34^ In this work, the analysis and characterisation of a putative TAm-long-chain acyl-[acyl-carrier-protein] reductase fusion PP_2782 (*Pp*KTFusion) and three of its thermophilic homologs (Tmar_2123 (*Tm*Fusion), ThesuDRAFT_00745 (*Ts*Fusion), and Tfer_2018 (*Tf*Fusion), from *Thermaerobacter marianensis*, *Thermaerobacter subterraneus*, and *Thermincola ferriacetica*, respectively) is described. The enzymes were cloned, truncated to their TAm domain, expressed, and tested *in vitro*. Results of the TAm activity screens, which were investigated qualitatively through colorimetric assays and quantitatively *via* reaction conversions, are presented. A more detailed account of the activity of *Pp*KTFusion and *Tf*Fusion (and their truncated TAm derivatives) at different pH levels, temperatures, amine donor and substrate concentrations, and with different solvents is also described.

## Results and discussion

### *Pp*KTFusion sequence analysis

**Fig. 3:**
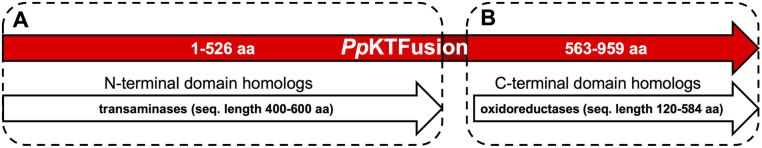
A visual representation of multiple sequence alignments of *Pp*KTFusion with its N- and C-terminal single domain homologs. *Pp*KTFusion alignment with (A) TAms, and (B) oxidoreductases returned by BLASTP analysis.

Protein Basic Local Alignment Search Tool (BLASTP) analysis was performed to identify and categorise *Pp*KTFusion homologs in order to investigate the TAm homology, relatedness within this fusion group, boundaries of the TAm domain, and to infer enzyme function of the partner protein. The search returned several groups of related sequences, which could be split into three tiers (Table S1, ESI†). Sequence length variability between entries prompted the use of a NCBI Conserved Domains tool, which identified two functional motifs within *Pp*KTFusion – (a) an AAT_I superfamily domain (PLP-dependent aspartate TAm (fold type I)), and (b) a COG5322 superfamily domain (predicted amino acid dehydrogenase) (Table S2, ESI†). Multiple sequence alignments (MSA) of the *Pp*KTFusion N- and C-terminal domain BLASTP results (limited to entries with single functional motifs) were further employed to determine boundaries of the two domains (Fig. 3). Based on these results, the N-terminal domain, between residues 1–526, was assigned as the TAm part, (Fig. 3(A)); the C-terminal domain between residues 563–959 (397 aa) was assigned the dehydrogenase/reductase part, (Fig. 3(B)); and residues between 527 and 562 (36 aa) were proposed to be the linker peptide.

### Gene cluster

Screening genomes of *Pp*KTFusion homologs showed that similar gene clusters (to PP_2777-PP_2787) were also present in many other organisms. Overall, 230 homologs with comparable gene clusters were identified (Fig. S1, ESI†). Most of these clusters were present in *Gammaproteobacteria* (predominantly in *Pseudomonas* and *Xenorhabdus* genera), two in *Betaproteobacteria*, and 23 in *Actinobacteria* (*Streptomyces* spp.), (Table 1).

**Table 1:** Bacterial lineage of species with comparable gene clusters

Phylum	Class	Order/genus/genome fragment
*Terrabacteria group*/*Actinobacteria* (23)	*Actinomycetia* (23)	*Streptomyces* (23)
*Proteobacteria* (206)	*Betaproteobacteria* (2)	Rhodocyclaceae (1), *Zoogloea* (1)
	*Gammaproteobacteria* (203)	*Aeromonas* (1), *Cellvibrio* (1), *Jejubacter* (1), *Kangiella* (1), *Klebsiella* (3), *Lysobacter* (2), *Photobacterium* (3), *Pseudoalteromonas* (5), *Pseudomonas* (160), *Rheinheimera* (1), *Vibrio* (2), *Xenorhabdus* (22), unknown (1)
	Unknown (1)	Unknown (1)
Unknown (1)	Unknown (1)	pEAF66 (1)

### *Pp*KTFusion phylogenetic analysis

**Fig. 4:**
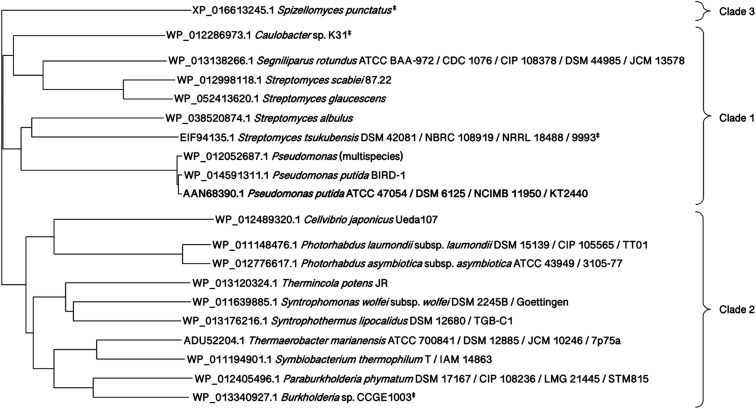
Phylogenetic tree of *Pp*KTFusion orthologs. Orthologs were identified by the OMA Browser, accessible at https://omabrowser.org/. The tree was constructed employing Neighbour-joining method using Clustal Omega MSA tool, accessible at https://www.ebi.ac.uk/Tools/msa/clustalo/. ‡Genome sequence not available.

To elucidate the enzyme's evolutionary pathway, phylogenetic analysis of the *Pp*KTFusion sequence was performed. Identification of orthologs is useful because enzyme function tends to be conserved in all species.^35^ A phylogenetic tree, constructed using *Pp*KTFusion orthologs, demonstrated that, aside from *Pseudomonas* spp., the most closely related homologs were found in *Streptomyces* spp. (clade 1, Fig. 4).

Several orthologs were also found in extremophiles, (clade 2, Fig. 4), indicating a broad distribution of this type of fusion enzyme. Investigation of gene neighbourhoods showed that more distant orthologs, including those in extremophiles, were located within different types of gene clusters (Table S4, ESI†).

Further analysis of the genomes of the three recruited thermophiles (as detailed below) using the antiSMASH online tool revealed the presence of NRP/PKS-type gene clusters likely encoding a prodiginine family-type alkaloid (Table S5, ESI†).

### Thermophilic homologs

Given the desirability for potential applications, three thermophilic TAm fusion homologs – Tmar_2123 from *Thermaerobacter marianensis* DSM 12885 (NCBI GenBank reference: ADU52204.1/WP_013496504.1) (*Tm*Fusion), ThesuDRAFT_00745 from *Thermaerobacter subterraneus* DSM 13965 (NCBI GenBank reference: EKP95021.1/WP_006903020.1) (*Ts*Fusion), and Tfer_2018 from *Thermincola ferriacetica* Z-0001 (NCBI GenBank reference: KNZ69380.1/WP_052218203.1) (*Tf*Fusion) – were selected for further study. *Tm*Fusion, *Ts*Fusion, *Tf*Fusion, and *Pp*KTFusion were aligned, and sequences of the thermophilic enzymes were divided into N- and C-terminal sections, in line with domain boundaries in *Pp*KTFusion (Fig. S2, Tables S6 and S7, ESI†). BLASTP of *Tm*Fusion, *Ts*Fusion, and *Tf*Fusion identified only a small number (1–10) of close homologs for each of the enzymes (Table S8, ESI†), providing little insight into their background. Overall, the distribution of the returned fusion-like di-domain entries showed a substantial overlap with *Pp*KTFusion BLASTP results.

### Structural homologs

Identification of structural homologs can also help determine the function and substrate range for the enzyme under the investigation, since many structurally similar enzymes (regardless of whether their primary sequences are homologous or not) tend to exhibit similar function.^36^

#### N-terminal domain homologs

Top entries included class-III TAms (EC 2.6.1) with sequence coverage >91%, (Tables S9 and S10, ESI†), supporting the proposition that the N-terminal domain in *Pp*KTFusion, *Tm*Fusion, *Ts*Fusion, and *Tf*Fusion enzymes was likely a PLP-dependent TAm. Among the top entries for the *Pp*KTFusion was a bifunctional enzyme fusion – diaminopelargonic acid TAm-dethiobiotin synthetase (DAPA AT-DTBS) from *Arabidopsis thaliana* (PDB: 4A0R), (Table S9, ESI†). DAPA AT (TAm) and DTBS (dethiobiotin synthetase) domains catalyse the second and third steps of a four-step biotin biosynthesis pathway. The monocistronic transcript producing DAPA AT-DTBS fusion originates from prokaryotic ancestor genes but is only found in plants and fungi.^17^ In bacteria, the same reactions are performed by separately encoded enzymes.^37^ In contrast to *Pp*KTFusion, the TAm domain in DAPA AT-DTBS fusion is positioned at the C-terminal.

For *Tm*Fusion, *Ts*Fusion, and *Tf*Fusion, one of the top structural homologs is PigE (PDB: 4PPM) – an ACOAT-like enzyme (Table S10, ESI†). This enzyme is involved in the biosynthesis of prodigiosin (red pigment) and, until recently, it was proposed to be a monofunctional enzyme responsible for the transamination of (*S*)-3-acetyloctanal.^38^ However, in light of the emergence of the AAR crystal structure (PDB: 6JZU), the function of PigE was reviewed and is now thought to include the reduction of a thioester intermediate to an aldehyde ((*S*)-3-acetyloctanal) as well as its subsequent transamination.^39–41^ Unlike in *Tm*Fusion, *Ts*Fusion, and *Tf*Fusion enzymes, the PigE TAm domain is located at the C-terminal of the fusion, suggesting convergent evolution. Another key structural homolog for thermophilic fusions is a thermostable TAm ω-TATR (PDB: 6IO1). It was proposed that at high temperatures the stability of the ω-TATR active site structure is preserved by the presence of a high mobility loop, which counteracts the effect of thermal perturbation.^42^

**Fig. 5:**
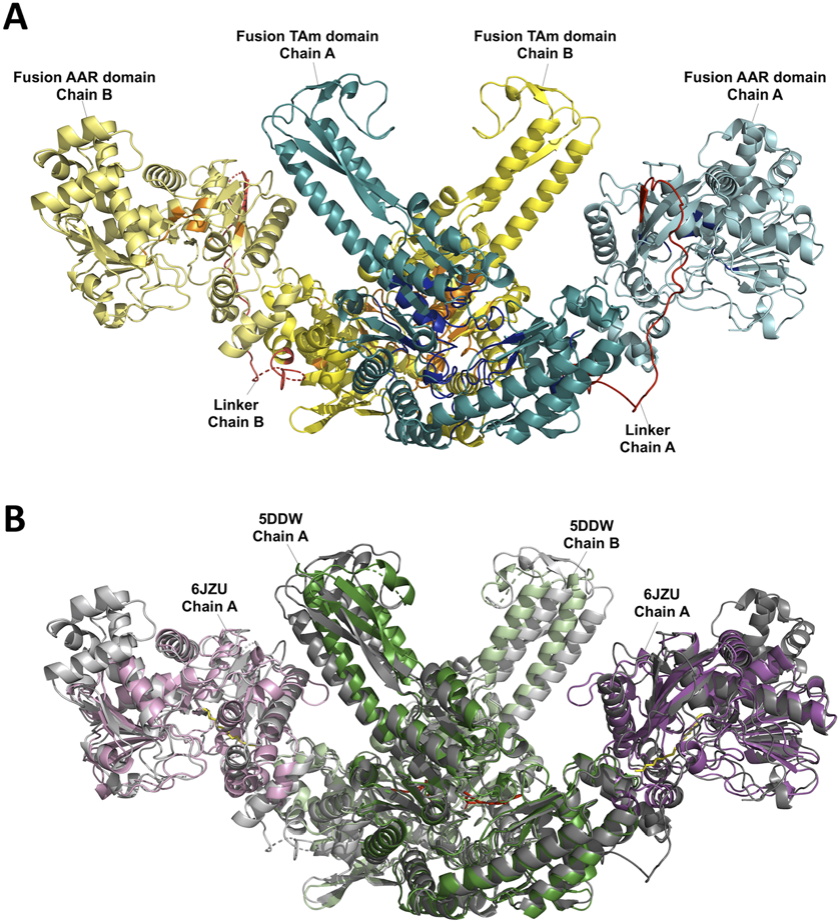
3D models of the transaminase fusion and its closest structural homologs. (A) Proposed quaternary structure of *Pp*KTFusion (assembled in PyMOL). Residues within the top 10% conservation scores are highlighted in dark blue for chain A (cyan) and orange for chain B (yellow); linker regions are depicted in red. (B) Structure of *Pp*KTFusion (grey) superimposed with its closest structural homologs – CrmG (PDB: 5DDW, chains A and B in green), and *Se*AAR (PDB: 6JZU, chain A in purple/pink).

Homologs CrmG (PDB: 5DDW) and YgjG (PDB: 5H7D) were among the top-ranking entries for both *Pp*KTFusion and the thermophilic set, indicating high structural similarity between all four enzymes under investigation, (see Fig. 5). CrmG is located within the caerulomycin A biosynthetic pathway, which is a hybrid PKS and NRPS system.^43^ Caerulomycin A, known to be present in *Streptomyces caeruleus* and *Actinoalloteichus cyanogriseus* WH1-2216-6, has been found to have antimicrobial and cytotoxic properties and is thus of great interest to the biomedical field. Another compound with a similar structure, collismycin A, was identified in *Streptomyces* spp.^44^ Monofunctional CrmG is responsible for the conversion of an aldehyde attached to a 2,2′-bipyridyl ring core structure to an amine, favouring l-glutamate and l-glutamine as amine donors.^43^ Finally, the YgjG homolog is from *Escherichia coli*. The conformation of its active site entrance is smaller and more hydrophobic compared to other class III TAms, explaining the preference for aliphatic diamine substrates.^45^ Previous analysis of the solved crystal structure of PigE C-terminal TAm domain also revealed its relatively small and hydrophobic active site.^46^ Although *Pp*KTFusion substrates for this study were initially proposed based on the gene cluster the enzyme was found in, the same range of compounds was extended to all four enzymes based on their structural similarity and analogy to PigE and YgjG.

#### C-terminal domain homologs

A long-chain acyl-[acyl-carrier-protein] reductase (AAR, PDB: 6JZU) from *Synechococcus elongatus* PCC 7942 was the only close match (at >75% residue alignment/coverage) to the *Pp*KTFusion, *Tm*Fusion, *Ts*Fusion, and *Tf*Fusion C-terminal region (Fig. 5, Tables S9 and S11, ESI†). After a structural alignment, the AAR active site catalytic residue C294 ^47^ was mapped onto C910, C909, C815, and C824 in the *Pp*KTFusion, *Tm*Fusion, *Ts*Fusion, and *Tf*Fusion C-terminal domain, respectively, (Table S12, ESI†). The structural alignment further revealed that the AAR region containing a highly conserved sequence ^*162*^GATGDIG^*168*^ for the NADP(H) binding (in the form GX_(1-3)_GX_(1-3)_G) was comparable in all four enzymes under the investigation (Table S12, ESI†). AAR (PDB: 6JZU) is responsible for the conversion of a long chain fatty acyl-ACP/fatty acyl-CoA to the corresponding aldehyde.^47^ The conversion proceeds *via* a ‘ping-pong’ mechanism through an acyl-enzyme intermediate as the acyl group covalently binds to C294 releasing ACP/CoA.^47–49^ In the subsequent step NADPH donates a hydride which enables release of the aldehyde from the enzyme.^47,49^ Interestingly, the *Pp*KTFusion, *Tm*Fusion, *Ts*Fusion, and *Tf*Fusion C-terminal domain primary sequence also aligned with the N-terminal domain of PigE.

### Expression of recombinant enzymes

For analysis *in vitro*, *Pp*KTFusion, *Tm*Fusion, *Ts*Fusion, and *Tf*Fusion were cloned into pET-28a(+), truncated to what was determined to be their TAm domain and expressed in *E. coli* BL21(DE3) or Rosetta 2(DE3) with a N-terminal His_6_-tag (Fig. S5–S8, ESI†). Enzymes were purified using immobilised metal affinity chromatography and then tested qualitatively using colorimetric assays and quantitatively *via* conversions determined by analytical HPLC.

### Transaminase activity screening

#### Colorimetric screening

**Fig. 6:**
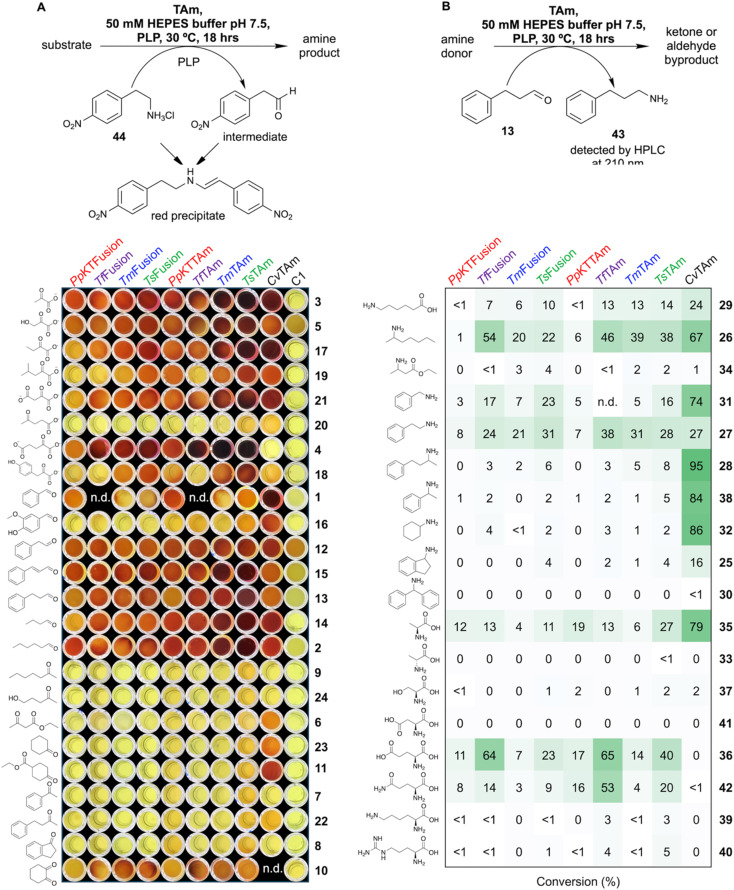
Transaminase activity screening of *Pp*KTFusion, *Tf*Fusion, *Tm*Fusion, *Ts*Fusion, *Pp*KTTAm, *Tf*TAm, *Tm*TAm, and *Ts*TAm, alongside *Cv*TAm control using 1–44. (A) Colorimetric assays with α-keto acids, aldehydes, and ketones employing 44 as an amine donor and shown with no enzyme (C1) control. Reactions were performed in triplicate or duplicate and contained 50 mM HEPES buffer at pH 7.5, 25 mM 44, 1.0 mM PLP, and a substrate at 10 mM. Reactions were initiated by the addition of enzyme (0.01 mg mL^−1^ final concentration) and run at 30 °C for 18 hours. The dark red colouration in samples indicates conversion levels ≥20%. (B) Conversions with various amine donors using 13 as a substrate. Assays were prepared with 25 mM amine donor, 10 mM 13, 1.0 mM PLP, and purified enzyme at 0.05 mg mL^−1^ and were performed in duplicate. Percentage conversions were determined using 43 yield detected at 210 nm by HPLC. Assays were run alongside no enzyme controls, and any background (if present) was subtracted from the reactions. Values shown are the mean of two measurements, with SD < 10%.

To establish transaminase activity, compounds 1–43 were used. The colorimetric assays employed 2-(4-nitrophenyl)ethan-1-amine 44 as an amine donor^50^ and aliphatic aldehydes and ketones as substrates. Initial results supported the *in silico* analysis that fusions and their truncated derivatives possess transaminase activity. All enzyme sets (*Pp*KTFusion/*Pp*KTTAm, *Tm*Fusion/*Tm*TAm, *Ts*Fusion/*Ts*TAm, and *Tf*Fusion/*Tf*TAm) showed good activity with a range of α-keto acid acceptors as well as with linear and unsubstituted aromatic aldehydes (Fig. 6(A)), suggesting such compounds could be among the native substrates. On the other hand, no activity, except with 1,2-cyclohexanedione 10, was detected with ketones, (Fig. 6(A)).

Variation in the range of compounds accepted by *Tm*Fusion/*Tm*TAm and *Ts*Fusion/*Ts*TAm, and *Tf*Fusion/*Tf*TAm and *Pp*KTFusion/*Pp*KTTAm see assays with 4-methyl-2-oxovalerate 19, and 4-hydroxyphenylpyruvate 18, (Fig. 6(A)), highlighted that the native substrates between these enzyme groups likely differ slightly. Although colorimetric assays were used for qualitative assessment, some additional inferences could also be made. Despite the difference in the concentration used between *Pp*KTFusion and *Pp*KTTAm (0.94 × 10^−7^ M and 1.66 × 10^−7^ M), the colour intensity in samples was not substantially dissimilar after 18 hours when using pyruvate as a substrate (Fig. S10, ESI†). Consequently, it was proposed that the difference in colorimetric readout between *Pp*KTFusion and *Pp*KTTAm in assays with 2-ketobutyrate 17, 4-methyl-2-oxovalerate 19, oxaloacetate 21, and butanal 14, (Fig. 6(A)), was due to structural differences between the fusion and its truncated derivative. In the case of *Pp*KTFusion (in samples with weaker colour intensity), the cited substrates could have had a more restricted access to the active site due to the presence of the C-terminal domain. Notably, unlike *Pp*KTTAm, where results were analogous, *Pp*KTFusion showed a better activity with hexanal compared to butanal, (Fig. 6(A)). Interestingly, such differences were not observed with thermophilic fusions and their truncated derivatives.

#### Use of HPLC analysis

To determine the type and the range of amine donors that could be utilised by the enzymes, 3-phenylpropionaldehyde 13 (as identified by colorimetric assays) was selected for experiments using HPLC analysis. As with the colorimetric assays, fusions and truncated TAms had a narrower substrate range compared to the *Cv*TAm control, and mainly showed a preference for l-glutamate 36, l-glutamine 42, l-alanine 35, and amines with aliphatic moieties, (Fig. 6(B)). Compared to *Pp*KTFusion/*Pp*KTTAm, the thermophilic enzymes accepted a broader range of amines, showing additional or greater activity with 6-aminohexanoic acid 29, 2-aminoheptane 26, 2-phenylethan-1-amine 27, benzylamine 31, 4-phenylbutan-2-amine 28 (all sets) and benzylamine 31, l-glutamate 36, and l-glutamine 42 (*Tf*Fusion/*Tf*TAm, *Ts*Fusion/*Ts*TAm). Interestingly, and unlike *Pp*KTFusion/*Pp*KTTAm, the thermophilic enzyme sets showed greater preference for l-glutamate 36 (similarly to PigE). Based on the range of accepted substrates, all tested fusions were proposed to be class III TAms.

### Characterisation of *Pp*KTFusion, *Tf*Fusion and their truncated derivatives *Pp*KTTAm, *Tf*TAm

The effect of pH, temperature, and co-solvent as well as the concentration of amine donor and substrate were investigated to establish the scope of *Pp*KTFusion/*Pp*KTTAm and *Tf*Fusion/*Tf*TAm transaminase activity. All assays employed 36 as an amine donor and 13 as the substrate/amine acceptor.

#### pH effect

**Fig. 7:**
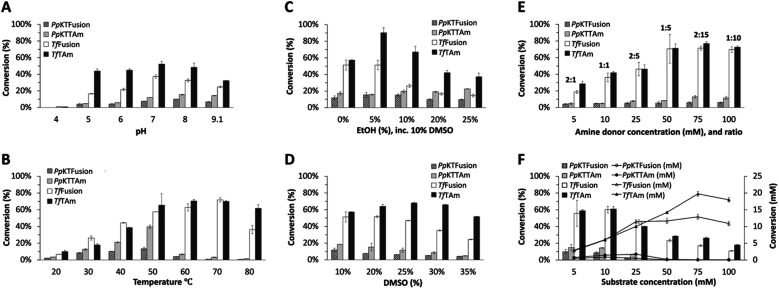
The effect of pH, temperature, solvent, and compound concentration on enzyme activity. Reactions contained: 1.0 mM PLP, purified enzyme at 0.05 mg mL^−1^, and (A) 50 mM (1) NaOAc buffer at pH 4.0, (2) NaOAc buffer at pH 5.0, (3) MES buffer at pH 6.0, (4) HEPES buffer at pH 7.0, (5) HEPES buffer at pH 8.0, (6) CHES buffer at pH 9.1, 20 mM 36, 10 mM amine 13, DMSO at 10% v v^−1^, and were performed at 30 °C (*Pp*KTFusion, *Pp*KTTAm) or at 50 °C (*Tf*Fusion, *Tf*TAm); (B) 50 mM HEPES buffer at pH 7.7, 20 mM 36, 10 mM 13, DMSO at 10% v v^−1^, and were performed at 20–80 °C degrees (at 10 °C degree steps) for all enzymes; (C and D) 50 mM HEPES buffer at pH 7.7, 20 mM 36, 10 mM 13, (1) DMSO at (a) 20%, (b) 25%, (c) 30%, (d) 35% v v^−1^; or (2) EtOH at (a) 5%, (b) 10%, (c) 20%, (d) 25% (with 10% DMSO, for substrate solubility), and were performed at 30 °C (*Pp*KTFusion, *Pp*KTTAm) or at 50 °C (*Tf*Fusion, *Tf*TAm); (E) 50 mM HEPES buffer at pH 7.7, 36 at (a) 5 mM, (b) 10 mM, (c) 25 mM, (d) 50 mM, (e) 75 mM, (f) 100 mM, 10 mM 13 (showing substrate to amine donor ratio), DMSO at 10% v v^−1^, and were performed at 30 °C (*Pp*KTFusion, *Pp*KTTAm) or at 50 °C (*Tf*Fusion, *Tf*TAm); (F) 50 mM HEPES buffer at pH 7.7, 13 at (a) 5 mM, (b) 10 mM, (c) 25 mM, (d) 50 mM, (e) 75 mM, (f) 100 mM, 2 equiv. 36, DMSO at 10% v v^−1^, and were performed at 30 °C (*Pp*KTFusion, *Pp*KTTAm) or at 50 °C (*Tf*Fusion, *Tf*TAm). All reactions were run for 18 hours and performed in triplicate or duplicate. Data is presented as the mean of three or two measurements, with error bars representing SD. The analysis was performed using the conversion of 13 to 43, by HPLC.

For both sets *Pp*KTFusion/*Pp*KTTAm and *Tf*Fusion/*Tf*TAm, enzyme activity was detected across a wide pH range. For *Pp*KTFusion/*Pp*KTTAm, greater product yield was observed at pH 8, whereas for *Tf*Fusion/*Tf*TAm, greater conversions were at pH 7, (Fig. 7(A)). For all enzymes, gradual reduction in conversion levels was noted at higher (>pH 8) and lower (<pH 7) pH, with a complete decline at pH 4, (Fig. 7(A)).

#### Effect of the reaction temperature

For *Pp*KTFusion and *Pp*KTTAm, an increase in conversion levels was observed at temperatures up to and including 50 °C, (Fig. 7(B)). At higher temperatures (>50 °C), there was a drop in performance for both enzymes, probably due to enzyme denaturation. In comparison, *Tf*Fusion showed an increase in conversion levels at temperatures up to and including 70 °C, (Fig. 7(B)). For *Tf*TAm, an increase in conversion levels was observed at temperatures of up to 50 °C, which was followed by a levelling-off between 50 and 70 °C, (Fig. 7(B)). Subjecting *Tf*Fusion to thermal treatment resulted in a decrease in residual activity to approx. 30% at 60 °C and 40% at 70 °C of the initial activity after two hours. Even without optimisation, this is a promising starting point, particularly given the absence of direct comparisons in the literature, (Fig. S11, ESI†).

#### Solvent effects

DMSO and ethanol are frequently added to enzyme assays for substrate solubilisation. To investigate solvent tolerance, a range of concentrations of each co-solvent was tested. For both *Pp*KTFusion and *Pp*KTTAm, enzyme activity was detected over a range of ethanol concentrations, with *Pp*KTTAm showing a slight increase in conversions at greater ethanol concentrations (tested in combination with 10% v v^−1^ DMSO), (Fig. 7(C)). In contrast, both *Tf*Fusion and *Tf*TAm showed a downward trend in conversion levels at >10% v v^−1^ of ethanol, (Fig. 7(C)).

With DMSO only, both *Pp*KTFusion and *Pp*KTTAm showed a gradual reduction in conversions with increasing solvent concentration, (Fig. 7(D)). For *Tf*Fusion, a gradual decrease in product yield was observed at >20% DMSO v.v^−1^. Interestingly, for *Tf*TAm the increase in DMSO concentration had only a marginal effect on performance, (Fig. 7(D)). Overall, these results indicated that ethanol, if suitable, would be a better co-solvent for *Pp*KTFusion/*Pp*KTTAm, and DMSO would work better with *Tf*Fusion/*Tf*TAm.

#### Amine donor concentration effect

Varying the concentration of 36 between 0.5 and 10 equivalents relative to 13 had little effect on the activity of *Pp*KTFusion, (Fig. 7(E)). For *Pp*KTTAm, a slight but gradual increase in conversions was observed at concentrations >10 mM but <75 mM (>1, but <7.5 equivalents), and a slight decrease – at >75 mM. The difference between the enzymes is thought to be due to the presence of the C-terminal domain. For *Tf*Fusion/*Tf*TAm, an increase in conversions was noted at up to 50 mM of 36 (0.5–5 equiv.); above that (5–10 equiv.) the activity remained comparatively unchanged, appearing to be saturating and producing no inhibition.

#### Substrate concentration effect

An increase in *Pp*KTFusion conversions (mM) was observed at concentrations of 13 up to 10 mM. For *Pp*KTTAm, the same was noted at up to 25 mM, (Fig. 7(F)). No activity was detected at ≥50 mM, for both enzymes (Fig. 7(F)). A drop in *Pp*KTFusion/*Pp*KTTAm activity at higher substrate concentrations (>25 mM) was indicative of substrate inhibition. This was validated by fitting data to substrate inhibition equations using non-linear regression analysis, which showed apparent enzyme inhibition even at micromolar substrate concentrations, (Table 2, Fig. S13, ESI†).

**Table 2:** Apparent kinetic parameters of *Pp*KTFusion, *Tf*Fusion and their truncated derivatives towards l-glutamate 36 and 3-phenylpropylaldehyde 13

Enzyme	*V* ^app^_max_ (nkatal mg^−1^)	*k* ^app^_cat_ (s^−1^) × 10^−2^	*K* ^app^_m_ (mM)	*k* ^app^_cat_/*K*^app^_m_ (s^−1^ mM^−1^) × 10^−2^	*K* ^app^_i_ (μM)
*Pp*KTFusion^a^	0.18	1.97	2.05	0.96	
*Pp*KTFusion^b^	n.d.	n.d.	n.d.	n.d.	3.37
*Pp*KTTAm^a^	0.37	2.23	9.85	0.23	
*Pp*KTTAm^b^	n.d.	n.d.	n.d.	n.d.	5.13
*Tf*Fusion^a^	2.65	26.72	16.44	1.63	
*Tf*Fusion^b^	11.61	117.10	50.61	2.31	52.92
*Tf*TAm^a^	2.47	12.25	9.57	1.28	
*Tf*TAm^b^	10.92	54.17	57.28	0.95	332.20

a l-Glutamate 36.

b 3-Phenylpropionaldehyde 13.

In contrast, a rapid increase in *Tf*Fusion performance was observed at substrate concentrations of up to 25 mM; this was followed by a more gradual improvement between 25 and 75 mM, (Fig. 7(F)). For *Tf*TAm, a gradual increase in activity was observed between 5 and 75 mM. At >75 mM, a slight drop in activity was noted for both enzymes, suggesting inhibition at greater concentrations (Fig. 7(F)). This, again, was supported by fitting data (Table 2, Fig. S13, ESI†).

Interestingly, following the truncation of *Pp*KTFusion, *K*^app^_m_, *k*^app^_cat_, and *V*^app^_max_ values for 36 increased, exhibiting lower affinity towards the amine donor but greater enzyme activity (Table 2). For 13, parameters could not be determined due to the apparent enzyme inhibition. In contrast, following the truncation of *Tf*Fusion, *k*^app^_cat_ values towards both 36 and 13 dropped by approx. two-fold, (Table 2). While the *K*^app^_m_ value for 36 decreased, it increased for 13, indicating an improved affinity towards the amine donor but a decreased affinity towards the acceptor, (Table 2). The changes were most likely due to the structural modification or absence of the C-terminal domain, which may have a role in amine donor and substrate binding, or possibly a combination of both.

### Comparison of enzyme parameters

On the whole, *k*_cat_ values of *Pp*KTFusion/*Pp*KTTAm and *Tf*Fusion/*Tf*TAm were relatively low compared to other known mesophilic^13,51^ and thermophilic^34,52^ TAms, respectively, but comparable to other structurally similar enzymes, (Table 3). This is unsurprising, considering that the main role of *Pp*KTFusion (and potentially *Tf*Fusion as well) is to facilitate the production of a secondary metabolite *in vivo*^20,23–25^ – where large quantities of the compound would likely be toxic to the cell. The two exceptions were the class III TAm (PDB: 3HMU), and the plant bifunctional DAPA AT-DTBS homolog. For the latter, *k*_cat_ was one order of magnitude lower than that of other enzymes, but in line with physiological requirements *in vivo*,^17^ whereas for the former, *k*_cat_ was one order of magnitude greater (Table 3). Although structurally similar to 3FCR, 3HMU was found to have different residues in its active site which, presumably, enhance its catalytic activity.^53^ Notably, the *k*_cat_ of the thermophilic homolog ω-TATR for pyruvate was comparable to that of *Tf*TAm. For (*S*)-MBA, the value was approx. three- and six-fold greater relative to *Tf*Fusion and *Tf*TAm, respectively. However, these parameters were determined at 60 °C, where the values were two- (for pyruvate) and three- (for (*S*)-MBA) fold greater than at 37 °C.^54^

**Table 3:** *Pp*KTFusion and *Tf*Fusion N-terminal domain turnover number comparison with key structural homologs

Enzyme	*k*_cat_ (s^−1^ × 10^−2^)	Organism	Source
**Mesophilic**			
*Pp*KTFusion^a^^,^^b^	1.97^c^	*Pseudomonas putida* KT2440	This work
*Pp*KTTAm^a^^,^^b^	2.23^c^	*Pseudomonas putida* KT2440	This work
DAPA AT-DTBS fusion (PDB: 4 A0R)^c^	0.12	*Arabidopsis thaliana*	17
DAPA AT^d^^,^^e^	1.70	*Mycobacterium tuberculosis*	56
DAPA AT^d^^,^^e^	1.30	*Escherichia coli*	37
Class III TAm (PDB: 3FCR)^f^	1.00	*Ruegeria* sp. TM1040	53
Class III TAm (PDB: 3HMU)^f^	38.0	*Ruegeria pomeroyi*	53
CrmG (PDB: 5DDW)^g^	0.80	*Actinoalloteichus* sp. WH1-2216-6	43

**Thermophilic**			
*Tf*Fusion^a^^,^^b^	117.10^h^/26.72^c^	*Thermincola ferriacetica* Z-0001	This work
*Tf*TAm^a^^,^^b^	54.17^h^/12.25^c^	*Thermincola ferriacetica* Z-0001	This work
ω-TATR (PDB: 6IO1)^f^	53.20^i^/72.30^j^	*Thermomicrobium roseum*	54

a Estimated *via* non-linear regression curve fitting using GraphPad Prism 9.

b Using 3-phenylpropionaldehyde 13 as a substrate and l-Glu 36 as an amine donor.

c Determined for 36.

d *A*. *thaliana* DAPA AT homolog.

e Using (*S*)-KAPA as a substrate and SAM as an amine donor (native substrates).

f Using pyruvate as a substrate and (*S*)-MBA as an amine donor.

g Using CRM M as a substrate and l-glutamine as an amine donor (native substrates).

h Determined for 13.

i Determined for pyruvate.

j Determined for (*S*)-MBA.

### Comparison of enzyme parameters

Although the activity of the C-terminal domains in fusions was not determined here, given their close structural similarity to AAR (PDB: 6JZU), their function is likely to be that of the AAR. The C-terminal domains of *Pp*KTFusion, *Tm*Fusion, *Ts*Fusion, and *Tf*Fusion were tested exhaustively for alcohol dehydrogenase activity with negative results. A homologous enzyme KecF (NCBI GenBank reference: WP_077570921.1; 80% ID/99% QC relative to *Pp*KTFusion) was proposed to perform a reduction of an ACP-polyketide intermediate to a polyketide aldehyde, with a subsequent transamination of the polyketide aldehyde to an amine.^25^ The suggestion was based on the analysis of an alkaloid generated *via* the homologous pathway in *P. koreensis*,^25^ (see Fig. 2). Due to the high sequence similarity between *Pp*KTFusion and KecF, enzyme function as well as substrate types are likely to be comparable (though KecF activity was not investigated *in vitro*). Importantly, PigE function was also recently reviewed, which led to the revision of MAP biosynthesis in the prodigiosin pathway.^41^ PigE (NCBI GenBank reference: WP_015376883.1), both a structural and primary sequence homolog with the opposite domain configuration (see above), is also thought to have a dual AAR-TAm function.^41^

**Fig. 8:**
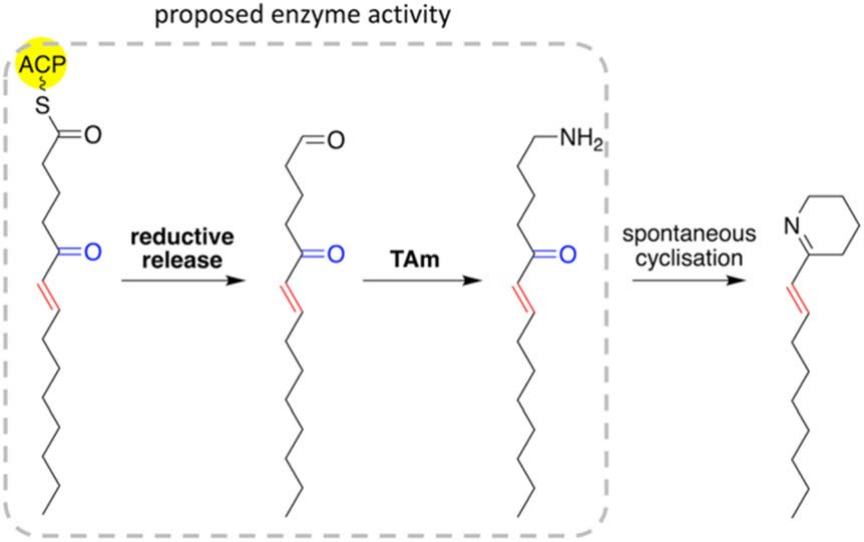
Proposed enzyme activity of *Pp*KTFusion, *Tm*Fusion, *Ts*Fusion and *Tf*Fusion. The highlighted substituent group in blue could have a hydroxyl configuration or might be absent; the highlighted aliphatic chain in red could be saturated or unsaturated, as are other parts of the tail.

Additional support for the TAm-AAR activity is afforded by the characterisation of *Pt*TAmH from *Pseudoalteromonas tunicata*. *Pt*TAmH is a di-domain enzyme with homology to all four fusions in this work: at 28–32% ID/89–98% QC. During the course of this work, *Pt*TAmH was found to be able to carry out the thioester reduction on ACP with subsequent transamination of aliphatic aldehyde substrates (Fig. 8). Importantly, the investigation also highlighted the preference of the enzyme's TAm domain for C_12_ aldehyde substrates.^55^

## Conclusions

The work presented herein provides analysis and the first known detailed account of the activity of four TAm fusions (from *P. putida* KT2440, and its thermophilic homologs from *T. marianensis*, *T. subterraneus* and *T. ferriacetica*) of this kind. All four enzymes showed preference for long/aliphatic aldehydes and a number of α-keto acids. The range of amine donors accepted was different between the enzyme sets. *Pp*KTFusion and its truncated derivative *Pp*KTTAm favoured the acceptance of l-alanine 35, l-glutamate 36, and l-glutamine 42 amino acids.

In contrast, the thermophilic enzymes also accepted aliphatic and aromatic non-amino acid compounds. Slight variation in substrate preference placed *PpKT*Fusion/*PpKT*TAm and *Tf*Fusion/*Tf*TAm, and *Tm*Fusion/*Tm*TAm and *Ts*Fusion/*Ts*TAm, in separate groups, and facilitated the selection of a thermophilic homolog for additional characterisation. A more detailed analysis of *Pp*KTfusion and the truncated *Pp*KTTAm indicated that activity of both enzymes was mostly comparable but, at certain parameters, including higher substrate/amine donor concentrations, or higher temperature, the activity of *Pp*KTTAm was enhanced – most likely owing to structural differences. However, truncation also affected enzyme affinity towards the amine donor l-glutamate 36. Due to the apparent enzyme inhibition by 3-phenylpropionaldehyde 13, a different substrate is recommended for further studies.

A more detailed analysis of *Tf*Fusion and *Tf*TAm transaminase activity has revealed the enzymes' remarkable ability to operate at elevated temperatures with catalytic rates approximately 13- and 6-fold greater than those of *Pp*KTfusion and *Pp*KTTAm, respectively. However, truncation had an overall negative impact on enzyme activity, resulting in a reduction in the turnover number as well as a decreased affinity towards the aldehyde substrate.

Currently, there are very few characterised thermophilic TAms,^5,33,34^ and none are known to possess bifunctional activity. Having established the transaminase activity, the future potential of these enzymes is significant. The enzymes could also be utilised for the conversion of activated fatty acids to amines, or more complex natural products.^55^ Additionally, requirement for the efficient alcohol to amine conversion could necessitate the evolution (or replacement) of the fusion C-terminal domain to acquire a carbonyl reductase/dehydrogenase activity. Due to their domain structure, long-chain acyl-[acyl-carrier-protein] reductases are thought to be potentially related to the short-chain dehydrogenase/reductase family; and, as the oxidoreductase scaffold is already in place, this would seem to be a plausible evolutionary route. The use of natural, and synthetic, fusions is a promising approach to improving existing biocatalytic routes for the production of industrially important bioactive molecules.

## Author contributions

L. P. performed the experimental work, processed the data, performed the analysis, and drafted the manuscript. The project was conceptualised and supervised by J. M. W. and H. C. H. The manuscript has been reviewed and edited by all contributing authors.

## Conflicts of interest

There are no conflicts to declare.

## Supplementary Material

RA-014-D3RA07081F-s001

## References

[cit1] Koszelewski D., Tauber K., Faber K., Kroutil W. (2010). Trends Biotechnol..

[cit2] Guo F., Berglund P. (2017). Green Chem..

[cit3] Höhne M., Bornscheuer U. T. (2009). ChemCatChem.

[cit4] Baud D., Jeffries J. W. E., Moody T. S., Ward J. M., Hailes H. C. (2017). Green Chem..

[cit5] Cárdenas-Fernández M., Sinclair O., Ward J. M. (2022). Microb. Biotechnol..

[cit6] Cosgrove S. C. , Brzezniak A., France S. P., Ramsden J. I., Mangas-Sanchez J., Montgomery S. L., Heath R. S. and Turner N. J., in Current Opinion in Chemical Biology, Elsevier Inc., 1st edn, 2018, vol. 37, pp. 131–149

[cit7] Gilio A. K., Thorpe T. W., Turner N., Grogan G. (2022). Chem. Sci..

[cit8] Mangas-Sanchez J., France S. P., Montgomery S. L., Aleku G. A., Man H., Sharma M., Ramsden J. I., Grogan G., Turner N. J. (2017). Curr. Opin. Chem. Biol..

[cit9] Wu S., Snajdrova R., Moore J. C., Baldenius K., Bornscheuer U. T. (2021). Angew. Chem., Int. Ed..

[cit10] Simić S., Zukić E., Schmermund L., Faber K., Winkler C. K., Kroutil W. (2022). Chem. Rev..

[cit11] Ingram C. U., Bommer M., Smith M. E. B., Dalby P. A., Ward J. M., Hailes H. C., Lye G. J. (2007). Biotechnol. Bioeng..

[cit12] Lichman B. R., Lamming E. D., Pesnot T., Smith J. M., Hailes H. C., Ward J. M. (2015). Green Chem..

[cit13] Villegas-Torres M. F., Martinez-Torres R. J., Cázares-Körner A., Hailes H., Baganz F., Ward J. (2015). Enzyme Microb. Technol..

[cit14] Skalden L., Peters C., Dickerhoff J., Nobili A., Joosten H.-J., Weisz K., Höhne M., Bornscheuer U. T. (2015). ChemBioChem.

[cit15] Wang Y., Tappertzhofen N., Méndez-Sánchez D., Bawn M., Lyu B., Ward J. M., Hailes H. C. (2019). Angew. Chem., Int. Ed..

[cit16] Jensen R. A. (1976). Annu. Rev. Microbiol..

[cit17] Cobessi D., Dumas R., Pautre V., Meinguet C., Ferrer J. L., Alban C. (2012). Plant Cell.

[cit18] Gross H., Loper J. E. (2009). Nat. Prod. Rep..

[cit19] Rabe K. S., Müller J., Skoupi M., Niemeyer C. M. (2017). Angew. Chem., Int. Ed..

[cit20] Martins dos Santos V. A. P. P. , Timmis K. N., Tümmler B. and Weinel C., in Pseudomonas, ed. J. Ramos, Springer US, Boston, MA., Boston, MA, 2004, pp. 77–112

[cit21] Nelson K. E., Weinel C., Paulsen I. T., Dodson R. J., Hilbert H., Martins dos Santos V. A. P., Fouts D. E., Gill S. R., Pop M., Holmes M., Brinkac L., Beanan M., DeBoy R. T., Daugherty S., Kolonay J., Madupu R., Nelson W., White O., Peterson J., Khouri H., Hance I., Chris Lee P., Holtzapple E., Scanlan D., Tran K., Moazzez A., Utterback T., Rizzo M., Lee K., Kosack D., Moestl D., Wedler H., Lauber J., Stjepandic D., Hoheisel J., Straetz M., Heim S., Kiewitz C., Eisen J. A., Timmis K. N., Düsterhöft A., Tümmler B., Fraser C. M., Lee P. C., Holtzapple E., Scanlan D., Tran K., Moazzez A., Utterback T., Rizzo M., Lee K., Kosack D., Moestl D., Wedler H., Lauber J., Stjepandic D., Hoheisel J., Straetz M., Heim S., Kiewitz C., Eisen J. A., Timmis K. N., Dusterhoft A., Tummler B., Fraser C. M., Chris Lee P., Holtzapple E., Scanlan D., Tran K., Moazzez A., Utterback T., Rizzo M., Lee K., Kosack D., Moestl D., Wedler H., Lauber J., Stjepandic D., Hoheisel J., Straetz M., Heim S., Kiewitz C., Eisen J. A., Timmis K. N., Düsterhöft A., Tümmler B., Fraser C. M. (2002). Environ. Microbiol..

[cit22] Chung E. J., Lim H. K., Kim J.-C., Choi G. J., Park E. J., Lee M. H., Chung Y. R., Lee S.-W. (2008). Appl. Environ. Microbiol..

[cit23] Kim J.-W., Kim E.-H., Kang Y.-S., Choi O.-H., Park C.-S. S., Hwang I.-G. (2006). J. Microbiol. Biotechnol..

[cit24] Davis E., Sloan T., Aurelius K., Barbour A., Bodey E., Clark B., Dennis C., Drown R., Fleming M., Humbert A., Glasgo E., Kerns T., Lingro K., McMillin M., Meyer A., Pope B., Stalevicz A., Steffen B., Steindl A., Williams C., Wimberley C., Zenas R., Butela K., Wildschutte H. (2017). Microbiologyopen.

[cit25] Lozano G. L., Park H. B., Bravo J. I., Armstrong E. A., Denu J. M., Stabb E. V., Broderick N. A., Crawford J. M., Handelsman J. (2019). Appl. Environ. Microbiol..

[cit26] Ye S., Braña A. F., González-Sabín J., Morís F., Olano C., Salas J. A., Méndez C. (2018). Front. Microbiol..

[cit27] Mullowney M., McClure R. A., Robey M. T., Kelleher N. L., Thomson R. J. (2018). Nat. Prod. Rep..

[cit28] Ye S., Molloy B., Braña A. F., Zabala D., Olano C., Cortés J., Morís F., Salas J. A., Méndez C. (2017). Front. Microbiol..

[cit29] Aron Z. D., Dorrestein P. C., Blackhall J. R., Kelleher N. L., Walsh C. T., Blackball J. R., Kelleher N. L., Walsh C. T. (2005). J. Am. Chem. Soc..

[cit30] Tillett D., Dittmann E., Erhard M., Von Döhren H., Börner T., Neilan B. A. (2000). Chem. Biol..

[cit31] Awodi U. R., Ronan J. L., Masschelein J., de los Santos E. L. C., Challis G. L. (2017). Chem. Sci..

[cit32] Ohno S., Katsuyama Y., Tajima Y., Izumikawa M., Takagi M., Fujie M., Satoh N., Shin-ya K., Ohnishi Y. (2015). ChemBioChem.

[cit33] Ferrandi E. E., Previdi A., Bassanini I., Riva S., Peng X., Monti D. (2017). Appl. Microbiol. Biotechnol..

[cit34] Márquez S. L., Atalah J., Blamey J. M. (2019). Enzyme Microb. Technol..

[cit35] Zahn-Zabal M., Dessimoz C., Glover N. M. (2020). F1000Research.

[cit36] Martin A. C., Orengo C. A., Hutchinson E. G., Jones S., Karmirantzou M., Laskowski R. A., Mitchell J. B., Taroni C., Thornton J. M. (1998). Structure.

[cit37] Eliot A. C., Sandmark J., Schneider G., Kirsch J. F. (2002). Biochemistry.

[cit38] Williamson N. R., Simonsen H. T., Ahmed R. A. A., Goldet G., Slater H., Woodley L., Leeper F. J., Salmond G. P. C. (2005). Mol. Microbiol..

[cit39] Kasparyan E., Richter M., Dresen C., Walter L. S., Fuchs G., Leeper F. J., Wacker T., Andrade S. L. A., Kolter G., Pohl M., Müller M. (2014). Appl. Microbiol. Biotechnol..

[cit40] Dresen C., Richter M., Pohl M., Lüideke S., Müller M. (2010). Angew. Chem., Int. Ed..

[cit41] Couturier M., Bhalara H. D., Monson R. E., Salmond G. P. C., Leeper F. J. (2021). RSC Chem. Biol..

[cit42] Kwon S., Lee J. H., Kim C. M., Jang H., Yun H., Jeon J.-H., So I., Park H. H. (2019). Sci. Rep..

[cit43] Zhu Y., Xu J., Mei X., Feng Z., Zhang L., Zhang Q., Zhang G., Zhu W., Liu J., Zhang C. (2016). ACS Chem. Biol..

[cit44] Kawatani M., Muroi M., Wada A., Inoue G., Futamura Y., Aono H., Shimizu K., Shimizu T., Igarashi Y., Takahashi-Ando N., Osada H. (2016). Sci. Rep..

[cit45] Cha H. J., Jeong J.-H., Rojviriya C., Kim Y.-G. (2014). PLoS One.

[cit46] Lou X., Ran T., Han N., Gao Y., He J., Tang L., Xu D., Wang W. (2014). Biochem. Biophys. Res. Commun..

[cit47] Gao Y., Zhang H., Fan M., Jia C., Shi L., Pan X., Cao P., Zhao X., Chang W., Li M. (2020). Nat. Commun..

[cit48] Lin F., Das D., Lin X. N., Marsh E. N. G. (2013). FEBS J..

[cit49] Warui D. M., Pandelia M. E., Rajakovich L. J., Krebs C., Bollinger J. M., Booker S. J. (2015). Biochemistry.

[cit50] Baud D., Ladkau N., Moody T. S. S., Ward J. M., Hailes H. C. C. (2015). Chem. Commun..

[cit51] Leipold L., Dobrijevic D., Jeffries J. W. E., Bawn M., Moody T. S., Ward J. M., Hailes H. C. (2019). Green Chem..

[cit52] Wang C., Tang K., Dai Y., Jia H., Li Y., Gao Z., Wu B. (2021). ACS Omega.

[cit53] Steffen-Munsberg F., Vickers C., Thontowi A., Schätzle S., Tumlirsch T., Svedendahl Humble M., Land H., Berglund P., Bornscheuer U. T., Höhne M., SvedendahlHumble M., Land H., Berglund P., Bornscheuer U. T., Höhne M. (2013). ChemCatChem.

[cit54] Mathew S., Deepankumar K., Shin G., Hong E. Y., Kim B. G., Chung T., Yun H. (2016). RSC Adv..

[cit55] Richardson S. M., Marchetti P. M., Herrera M. A., Campopiano D. J. (2022). ACS Catal..

[cit56] Mann S., Ploux O. (2006). FEBS J..

