# Supplementary material for: Natural transaminase fusions for biocatalysis
Source: RSC Adv. 2024 Jan 31;14(6):4264–73. doi: 10.1039/d3ra07081f (PMC10829540; doi:10.1039/d3ra07081f)
Supplement: RA-014-D3RA07081F-s001 [file RA-014-D3RA07081F-s001.pdf]

Electronic Supplementary Material (ESI) for RSC Advances.
This journal is © The Royal Society of Chemistry 2024

Supporting Information

Natural transaminase fusions for biocatalysis

Luba Prout,[a] Helen C. Hailes[b] and John M. Ward[*a]

Table of Contents

Table of Contents 2

Table of Tables 3

Table of Figures 3

1. Experimental 4

1.1 Bioinformatics 4
1.1.1 BLASTP of the *Pp*KTFusion (1-959 aa) 4
1.1.2 Conserved domains 6
1.1.3 MSA 6
1.1.4 BLASTP of the *Pp*KTFusion N- (1-526 aa) and C-terminal (527-959 aa) domains 6
1.1.5 *P. putida* KT2440 gene cluster analysis 8
1.1.6 Phylogenetic analysis 8
1.1.7 Thermophilic homologs 12
1.1.8 Residue conservation 15
1.1.9 Structural homologs 16
1.1.10 3D Models 20
1.1.11 Structural alignment 21

1.2 Protein expression and purification 22
1.2.1 Protein expression – general protocol 22
1.2.2 Lysate preparation by sonication 22
1.2.3 Enzyme purification 22

1.3 Enzyme assays 27
1.3.1 Colorimetric assays with purified enzymes 27
1.3.2 End-point assays 27
1.3.3 Thermostability assays 29

1.4 Analytical HPLC 29

1.5 Kinetic parameters of *Pp*KTFusion/*Pp*KTTAm and *Tf*Fusion/*Tf*TAm 30

2. Protein sequences 31

2.1 Pseudomonas putida KT2440 PP_2782 31
2.1.1 *Pp*KTFusion protein sequence 31
2.1.2 *Pp*KTTAm protein sequence 31
2.1.3 *Pp*KTDHR protein sequence 31

2.2 Thermaerobacter marianensis DSM 12885 Tmar_2123 32
2.2.1 *Tm*Fusion protein sequence 32
2.2.2 *Tm*TAm protein sequence 32
2.2.3 *Tm*DHR protein sequence 32

2.3 Thermaerobacter subterraneus DSM 13965 ThesuDRAFT_00745 33
2.3.1 *Ts*Fusion protein sequence 33
2.3.2 *Ts*TAm protein sequence 33
2.3.3 *Ts*DHR protein sequence 33

2.4 Thermincola ferriacetica Z-0001 Tfer_2018 34
2.4.1 *Tf*Fusion protein sequence 34
2.4.2 *Tf*TAm protein sequence 34
2.4.3 *Tf*DHR protein sequence 34

3. References 35

Table of Tables

Table S1. *Pp*KTFusion BLASTP results summary. 5
Table S2. *Pp*KTfusion conserved domain general function prediction by NCBI CD-Search. 6
Table S3. *Pp*KTFusion C-terminal domain BLASTP results summary. 7
Table S4. *Pp*KTFusion KT2440 natural fusion OMA-identified orthologs with gene neighbourhood. 9
Table S5. antiSMASH output for the selected thermophilic strains. 11
Table S6. Thermophilic fusion sequence similarity results. 13
Table S7. Thermophilic fusion conserved domain' general function prediction by NCBI CD-Search. 13
Table S8. Thermophilic fusion BLASTP results summary. 14
Table S9. *Pp*KTFusion N- and C-terminal domain principal structural homologs. 17
Table S10. Thermophilic enzyme N-terminal domain principal structural homologs. 18
Table S11. Thermophilic enzyme C-terminal domain principal structural homologs. 19
Table S12. Results of *Pp*KTFusion conserved residue mapping to AAR and thermophilic homologs through a 3D structure alignment. 21

Table of Figures

Fig. S1. Phylogenetic tree showing all available *Pp*KTFusion homologs with comparable gene clusters (as at January 2022). 8
Fig. S2. *Pp*KTFusion and thermophilic homolog' amino acid sequence alignment. 12
Fig. S3. *Pp*KTFusion residue conservation analysis. 15
Fig. S4. 3D models of the *P. putida* KT2440 transaminase fusion and its thermophilic homologs. 20
Fig. S5. SDS-PAGE of *Pp*KTFusion, *Pp*KTTAm, and *Pp*KTDHR expression, post-optimisation. 23
Fig. S6. SDS-PAGE gel of *Tm*Fusion, *Tm*TAm, and *Tm*DHR expression 24
Fig. S7. SDS-PAGE gel of *Ts*Fusion, *Ts*TAm, and *Ts*DHR expression. 25
Fig. S8. SDS-PAGE gel of *Tf*Fusion, *Tf*TAm, and *Tf*DHR expression 26
Fig. S9. Typical 3-phenyl-1-propylamine HPLC standard curve used to determine product concentration in samples. 27
Fig. S10. Colour intensity development in control samples 28
Fig. S11. Thermostability analysis of *Tf*Fusion. 29
Fig. S12. Analytical HPLC method. 29
Fig. S13. Non-linear regression plots of *Pp*KTFusion/*Pp*KTTAm and *Tf*Fusion/*Tf*TAm 30
Fig. S14. *Pp*KTFusion protein sequence 31
Fig. S15. *Pp*KTTAm protein sequence 31
Fig. S16. *Pp*KTDHR protein sequence 31
Fig. S17. *Tm*Fusion protein sequence 32
Fig. S18. *Tm*TAm protein sequence 32
Fig. S19. *Tm*DHR protein sequence 32
Fig. S20. *Ts*Fusion protein sequence 33
Fig. S21. *Ts*TAm protein sequence 33
Fig. S22. *Ts*DHR protein sequence 33
Fig. S23. *Tf*Fusion protein sequence 34
Fig. S24. *Tf*TAm protein sequence 34
Fig. S25. *Tf*DHR protein sequence 34

1. Experimental

1.1 Bioinformatics

1.1.1 BLASTP of the *Pp*KTFusion (1-959 aa)

To search for natural fusion primary sequence homologs, an amino acid sequence of an enzyme was used as input for BLASTP (NCBI), accessible at https://blast.ncbi.nlm.nih.gov/Blast.cgi?PAGE=Proteins. Default parameters were used throughout, except for the maximum number of sequences, which was increased to 1000.

Homologs with >99% query cover (QC) and >72% ID were only found in species of *Pseudomonas* genus. These were class III PLP-dependent TAm enzymes with sequence lengths of 955 aa, 956 aa, and 959 aa. Sequences with 69-99% QC and 24-46% identity (ID) were identified in various genera – but predominantly in *Bacillus*, *Leptospira*, *Nocardia*, *Photorhabdus*, *Pseudomonas*, *Rhodococcus*, *Streptomyces*, and *Xenorhabdus* (**Table S1**). Entries with 21-71% QC and 24-44% ID were from various genera and were primarily between 400-600 aa in length and included enzymes classified as class III PLP-dependent TAms, notably acetylornithine, putrescine or succinyldiaminopimelate TAms, or hypothetical proteins.

Table S1. *Pp*KTFusion BLASTP results summary.

Number of sequences	Query cover	Identity	Genus and Species
**Tier 1**			
64 fusion-like[a]	99-100%	95-100%	*Pseudomonas putida* group, *Pseudomonas* sp., including 43(2021) / B8(2017) / B14(2017) / C5pp / CFA / JY-Q / Leaf58 / NBRC 111118 / NBRC 111125 / SID14000 / SWI36 / SWRI50 / p21 / XWY-1, *Pseudomonas alloputida*, *Pseudomonas asiatica*, *Pseudomonas monteilii*, *Pseudomonas plecoglossicida*, *Pseudomonas taiwanensis* SJ9, *Pseudomonas tehranensis*, *Aeromonas caviae**
117 fusion-like[a]	99-100%	80-83%	*Pseudomonas asgharzadehiana*, *Pseudomonas asplenii*, *Pseudomonas baetica*, *Pseudomonas bananamidigenes*, *Pseudomonas botevensis*, *Pseudomonas caspiana*, *Pseudomonas crudilactis*, *Pseudomonas ekonensis*, *Pseudomonas fluorescens*, *Pseudomonas glycinae*, *Pseudomonas jessenii*, *Pseudomonas koreensis*, *Pseudomonas leptonychotis*, *Pseudomonas mandelii*, *Pseudomonas marginalis*, *Pseudomonas monsensis*, *Pseudomonas prosekii*, *Pseudomonas sivasensis*, *Pseudomonas* spp., including 22E 5 / 2822-17 / 31-12 / 31E 6 / 43NM1 / A-RE-26 / AD21 / B11(2017) / B14-6 / B15(2017) / B26(2017) / B28(2017) / B35(2017) / CFBP13506 / F01002 / FIT28 / FSL W5-0299 / GM / IAC-BECa141 / Irchel 3H9 / Irchel s3a12 / Irchel s3f10 / Irchel s3h9 / LG1E9 / MG-9 / MIACH / MS586 / MWU318 / NIBRBAC000502773 / ok266 / PGPPP2 / R16(2017) / R84 / RIT357 / Root329 / RU47 / S49 / SC3(2021) / SCA2728.1_7 / SH10-3B / SJZ131 / SW-3, *Pseudomonas stutzeri*, *Pseudomonas tensinigenes*, *Pseudomonas viridiflava*, *Pseudomonas zeae*
		72-74%	*Atopomonas hussainii* (formerly *Pseudomonas hussainii*), *Pseudomonas leptonychotis*, *Pseudomonas* sp. CC6-YY-74 / OF001 / A-1 / MAHUQ-58 / SDI
		68%	*Unidentified Gammaproteobacteria bacterium*
**Tier 2**			
580 fusion-[a] or large multi-domain-[b] like	69-99%	24-46%	various species, predominantly from *Bacillus*, *Deltaproteobacteria*, *Firmicutes*, *Gemmatimonadetes*, *Leptospira*, *Micromonospora*, *Nocardia*, *Photorhabdus*, *Planctomycetes*, *Pseudoalteromonas*, *Pseudomonas*, *Rhodococcus*, *Streptomyces*, *Syntrophomonadaceae*, and *Xenorhabdus* genera
**Tier 3**			
948 TAm-like[c]	21-71%	24-44%	various species, predominantly from *Actinosynnema*, *Bacillus*, *Brevibacillus*, *Buttiauxella*, *Citrobacter*, *Cronobacter*, *Desulfotomaculum*, *Dickeya*, *Enterobacter*, *Enterobacteriaceae*, *Escherichia*, *Kitasatospora*, *Klebsiella*, *Mesorhizobium*, *Micromonospora*, *Ochrobactrum*, *Pectobacterium*, *Pseudomonas*, *Rhizobium*, *Rhodococcus*, *Saccharothrix*, *Shigella*, *Streptomyces* genera

[a] entries with sequence length ranging between 720-1200 aa

[b] entries with sequence length >1400 aa

[c] entries with sequence length ranging between 200-600 aa

*more recent searches (performed for keeping up-to-date with the emerging new data) have now also identified a close homolog in *Aeromonas caviae*.

1.1.2 Conserved domains

Conserved domains within the *P. putida* transaminase fusion (*Pp*KTFusion) were identified using NCBI CD-Search, accessible at https://www.ncbi.nlm.nih.gov/Structure/cdd/wrpsb.cgi.

Table S2. *Pp*KTfusion conserved domain general function prediction by NCBI CD-Search.

Enzyme	Conserved Domain	aa region
*Pp*KTFusion<br>AAN68390.1	AAT_I superfamily<br>PLP-dependent aspartate aminotransferase (fold type I)	41-521
	COG5322 superfamily<br>Predicted amino acid dehydrogenase [general function prediction only]	610-948

1.1.3 MSA

Multiple sequence alignments (MSA), comprised of natural fusion (or parts thereof) primary sequence homologs, were generated using ClustalOmega online tool (EMBL-EBI), accessible at http://www.ebi.ac.uk/Tools/msa/clustalo/. Protein sequence alignment results were visualised using Sequence Manipulation Suite, accessible at https://www.bioinformatics.org/sms2/index.html.

1.1.4 BLASTP of the *Pp*KTFusion N- (1-526 aa) and C-terminal (527-959 aa) domains

Additional BLASTP analysis of the proposed N-terminal domain (1-526 aa) returned analogous results to that generated by *Pp*KTFusion itself. In contrast, BLASTP results of the proposed C-terminal domain (527-959 aa) were more varied and included entries that were not returned by the *Pp*KTFusion search (**Table S3**). Of these, over 200 entries were dehydrogenase-/reductase-like (including 123 shikimate/quinate 5-dehydrogenases, 18 semialdehyde dehydrogenases, 17 amino acid dehydrogenases, 4 glutamyl-tRNA reductases, and 3 long-chain acyl-carrier-protein reductases) with the sequence length ranging between 120-584 aa. All entries with the single functional motif aligned with the 551/561-959 aa region of *Pp*KTFusion (results not shown).

Table S3. *Pp*KTFusion C-terminal domain BLASTP results summary.

Number of sequences	Query cover	Identity	Genus and Species
**Tier 1**			
17 fusion-like[a]	99-100%	80-100%	*Pseudomonas*, *Pseudomonas mandelii*, *Pseudomonas monteilii*, *Pseudomonas putida*, *Pseudomonas* sp. B11(2017) / B14(2017) / B15(2017) / B26(2017) / B28(2017) / B35(2017) / B8(2017) / MIACH / R16(2017)
**Tier 2**			
180 fusion-[a] or large multi-domain-[b] like	66-99%	20-49%	various species, predominantly from *Anaerolineae*, *Anaerolineales*, *Armatimonadetes*, *Chloroflexi*, *Firmicutes*, *Pseudomonas* and *Rhodococcus* genera
364 dehydro-genase-like and other[c]	52-94%	20-42%	various species, predominantly from *Anaerolineaceae*, *Armatimonadetes*, *Caldanaerobacter*, *Cellulomonas*, *Desulfotomaculum*, *Moorella*, *Omnitrophica*, *Peptococcaceae*, *Thermoanaerobacter*, *Thermoanaerobacterium* genera
**Tier 3**			
8 fusion-like[a]	24-49%	27-32%	predominantly from *Vibrio* genera
25 dehydro-genase-like and other[c]	9-44%	25-41%	various

[a] entries with sequence length ranging between 680-980 aa

[b] entries with sequence length >1500 aa

[c] entries with sequence length ranging between 200-580 aa

1.1.5 *P. putida* KT2440 gene cluster analysis

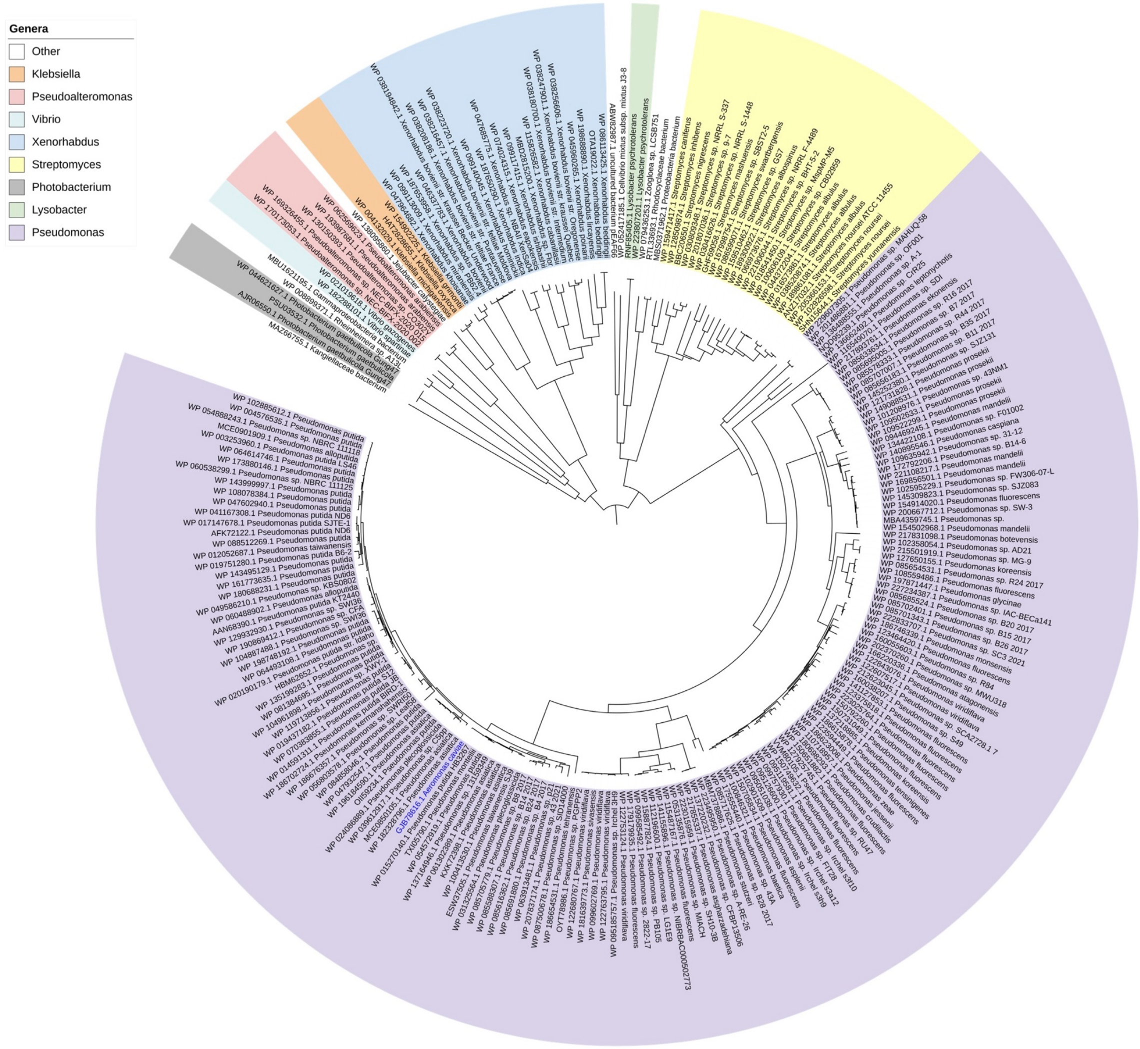

Fig. S1. Phylogenetic tree showing all available *Pp*KTFusion homologs with comparable gene clusters (as at January 2022). The tree was generated using https://itol.embl.de/.

1.1.6 Phylogenetic analysis

*Pp*KTFusion (PP_2782), *Tm*Fusion (Tmar_2123), *Ts*Fusion (ThesuDRAFT_00745), and *Tf*Fusion (Tfer_2018) gene orthologs and local synteny were analysed using OMA (orthologous matrix) Browser, accessible at https://omabrowser.org/.

Table S4. *Pp*KTFusion KT2440 natural fusion OMA-identified orthologs with gene neighbourhood.

Organism / Gene neighbourhood[a]	Ortholog NCBI Reference	**Organism Properties** *Morphology*: Gram, shape, motility. *Environment*: physico-chemical properties, oxygen, temp.	Seq. length (aa)	% ID
Pseudomonas putida KT2440 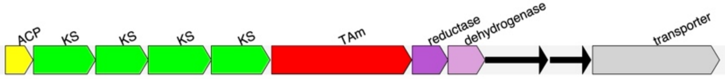	AAN68390.1	Gram-negative, rod-shaped, motile, aerobic, mesophilic	959	ref
Pseudomonas putida BIRD-1 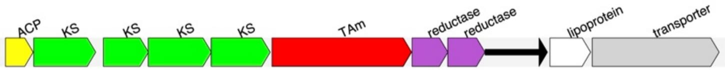	WP_014591311.1	Gram-negative, rod-shaped, motile, aerobic, mesophilic	956	99%
Pseudomonas taiwanensis 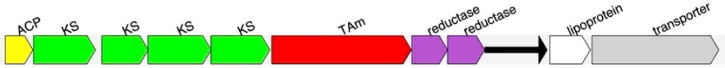	WP_012052687.1	Gram-negative, rod-shaped, motile; non-halophilic, aerobic, mesophilic	956	98%
Streptomyces albulus 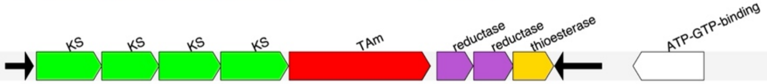	WP_038520874.1	Gram-positive, filamentous, nonmotile; aerobic, mesophilic	967	45%
Streptomyces scabiei 87.22 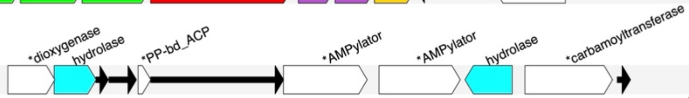	WP_012998118.1	Gram-positive, filamentous/tailed, motile; aerobic, mesophilic	910	30%
Streptomyces glaucescens 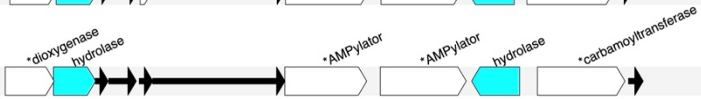	WP_052413620.1	Gram-positive, spore-forming, aerobic, mesophilic	906	31%
Photorhabdus laumondii subsp. laumondii 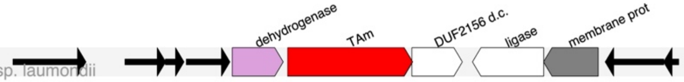	WP_011148476.1	Gram-negative, rod-shaped, motile, facultative anaerobe, mesophilic	855	25%
Photorhabdus asymbiotica 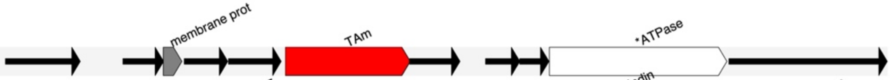	WP_012776617.1	Gram-negative, rod-shaped, nonmotile, facultative anaerobe, mesophilic	855	25%
Cellvibrio japonicus Ueda107 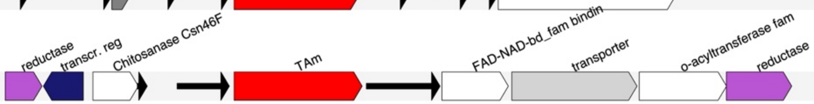	WP_012489320.1	Gram-negative, rod-shaped, motile, aerobic, mesophilic	876	26%

Table S4. ***Continued*****.**

Organism / Gene neighbourhood[a]	Ortholog NCBI Reference	Organism Properties<br>*Morphology*: Gram, shape, motility.<br>*Environment*: physico-chemical properties, oxygen, temp.	Seq. length (aa)	% ID
Paraburkholderia phymatum STM815 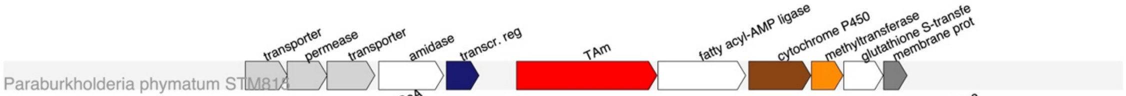	WP_012405496.1	Gram-negative, rod-shaped, nonmotile, non-halophilic, aerobic, mesophilic	955	29%
Syntrophomonas wolfei subsp. wolfei str. Goettingen G311 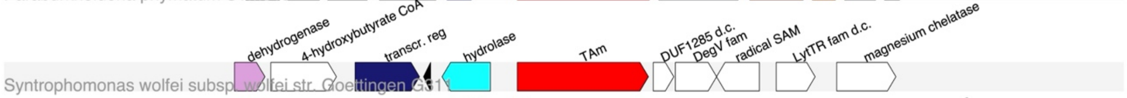	WP_011639885.1	Gram-negative, spirilla, nonmotile; anaerobic, mesophilic	891	30%
Syntrophothermus lipocalidus DSM 12680 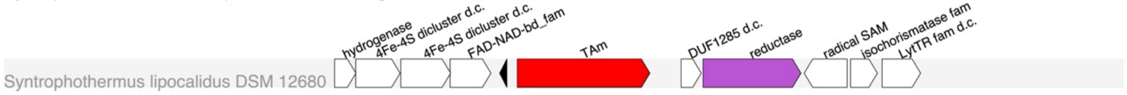	WP_013176216.1	Gram-positive, rod-shaped, nonmotile; anaerobic, thermophilic	901	31%
Thermincola potens JR 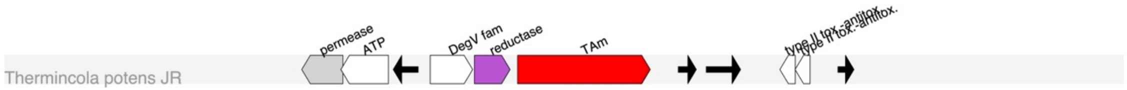	WP_013120324.1	Gram-positive, rod-shaped, nonmotile; anaerobic, thermophilic	902	29%
Thermaerobacter marianensis DSM 12885 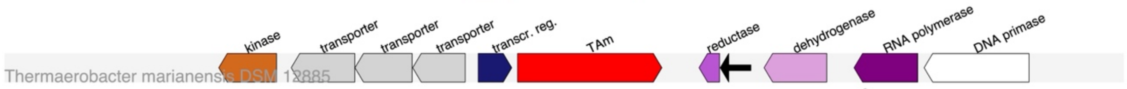	ADU52204.1/ WP_013496504.1	Gram-positive; Gram-negative at stationary phase, rod-shaped, nonmotile; halophilic, aerobic, hyperthermophilic	981	28%
Symbiobacterium thermophilum IAM 14863 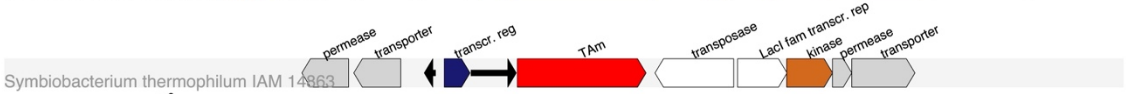	WP_011194901.1	Gram-positive, rod-shaped, nonmotile; non-halophilic, microaerophilic, thermophilic	875	30%
Segniliparus rotundus DSM 44985 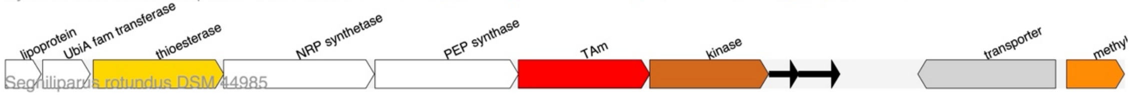	WP_013138266.1	Gram-positive, rod-shaped, nonmotile; aerobic, mesophilic	894	29%

ACP-like protein; oxidoreductase / dehydrogenase family protein; hydrolase family protein; transcription regulator protein; hypothetical protein; thioesterase; methyltransferase; 3-oxoacyl-(ACP) synthase II-like protein / beta-ketoacyl synthase-like protein; PLP-dependent TAm; kinase; transport or membrane protein; other; cytochrome 450; RNA polymerase

ACP: acyl carrier protein; KS: 3-oxoacyl-ACP synthase/β-ketoacyl synthase; TAm: transaminase; transcr. reg.: transcription regulation enzyme; *predicted; d.: domain; d.c.: domain-containing; fam.: family. Gene neighbourhood was visualised using a Python script ('GeneNBHD'), with ORF data pulled directly from NCBI.

Table S5. antiSMASH output for the selected thermophilic strains.

Compound Type	Most similar known cluster		Known cluster product description	Ref	Similarity %
Thermaerobacter marianensis DSM 12885					
saccharide[a]	fengycin	NRP	antimicrobial lipopeptide/fungicide	1	13%
fatty acid[b]	undecylprodigiosin	NRP + polyketide	prodiginine family alkaloid with antifungal properties and potential antimalarial, anticancer, immunosuppressive and antialgal activity	2	9%
saccharide[a]	burkholderic acid	NRP + polyketide (modular type I)	a signalling molecule	3	6%
saccharide[a] (x5)	none found				
***Thermaerobacter subterraneus* DSM 13965**					
saccharide[a]	fengycin	NRP	see above	1	13%
fatty acid[b]	undecylprodigiosin	NRP + polyketide	see above	2	9%
saccharide[a] (x7)	none found				
***Thermincola ferriacetica* Z-0001**					
saccharide[a]	L-2-amino-4-methoxy-trans-3-butenoic acid	NRP	non-proteinogenic amino acid toxin with antibacterial and anticancer properties	4,5	20%
saccharide[a]	S-layer glycan	saccharide	*G. stearothermophilus* cell surface protein polysaccharide	6	20%
saccharide[a]	legionaminic acid	other	an amine-containing monosaccharide (sialic acid analogue) present on the surface of bacterial pathogens	7	16%
fatty acid[b]	undecylprodigiosin	NRP + polyketide	see above	2	9%
saccharide[a]	paulomycin	other	glycosylated antibiotic	8	3%
cyclic-lactone-autoinducer[c]	7-deoxypactamycin	polyketide (iterative type I) + saccharide (hybrid/tailoring)	antitumor antibiotic	9	3%
saccharide[a] (x7)	none found				
cyclic-lactone-autoinducer[c]	none found				
β-lactone[d]	none found				
ranthipeptide[e]	none found				
halogenated[f]	none found				

[a] saccharide cluster (likely from primary metabolism)
[b] fatty acid cluster (likely from primary metabolism)
[c] agrD-like cyclic lactone autoinducer peptide
[d] beta-lactone (cyclic carboxylic ester) containing protease inhibitor
[e] cysteine-rich / non-α thioether-containing RiPP peptide
[f] cluster containing a halogenase (potentially generating a halogenated product)

1.1.7 Thermophilic homologs

```
PpKTFusion/1-959 MTVMDYRDFVRPKFVGLLQALGLECEFERALGSQLFYRNPKGDMVTVTDFLGGYGAALFGHNDPQFVDQLCALLRSDVPF 80
TmFusion/1-981   M--HPFRQYVNPHLGELLEQIQMDKRFVRGQGCWLWDED----GRRYLDFVAAYGALPFGFNPPEIWAALEEARRQGEPS 74
TsFusion/1-886   M--HPFRQYVNPHLAELLEQIQMDKRFVRGEGCWLWDDQ----GRRYLDFVAAYGALPFGFNPPEIWAALEEARHRGEPS 74
TfFusion/1-902   ----MKFSSLNPTMDKLFECFKLDVAYVKGEGTYLYDQA----GNKYLDFIAQYGAVPFGYNPPELVAAAKKYFDLSLPS 72

PpKTFusion/1-959 NAQMSIRGAAGQLGRALSDAFNRELKNTERYISTFSNSGAEAVEIAVKHAEFRRQKSLQKQFDDIDFTLASLTASEHAYR 160
TmFusion/1-981   FIQPSFLNAAGELARRLIEVA-PPG----LRYVTFANSGAEAVEAAIKLARA---------A------------------ 122
TsFusion/1-886   FVQPSFLNAAGELARRLIEVA-PPG----LRYVTFANSGAEAVEAAIKLARA---------A------------------ 122
TfFusion/1-902   MVQPSIPVKAVELAEMLLQLA-PGE----MAQATFCQSGAEAVEVAIKLARS---------T------------------ 120

PpKTFusion/1-959 ELDVADLDLPAGVLPATLNSVTLRQVVEAVRQHNLAQLHIEPVFVALRGSFHGKLVNTVQLTYGRQYRAPFARFGLNVEF 240
TmFusion/1-981   ---------------------T-----------------GRPRILSTENAFHGKTFGALSATHRAAYQDVFFAPAEGFDK 164
TsFusion/1-886   ---------------------T-----------------GRPRILSTDNGFHGKTFGALSATHRAAYQDAFFAPAEGFDK 164
TfFusion/1-902   ---------------------T-----------------GKPKILSTKNSFHGKTMGALSATGRDVYQKPFFTPVPGFEH 162

PpKTFusion/1-959 IDPQQPHQLQELP--ARHTHHWLSLQWDGEHLHVLQLPFSAITAVLMEPIQGEGGINEFAAEFYLGLRKLCNEQQCPLVV 318
TmFusion/1-981   VPYGDVAALEQALAA----------------------RPGEYAAFLVEPIQGEGGIVVPPPGYLRAAREICRRHGVLFIV 222
TsFusion/1-886   VPYGDLEALERALAA----------------------RPGEYAAFLVEPIQGEGGIVVPPPGYLREAREVCRRHGVLFIA 222
TfFusion/1-902   IPFGDLDMLENKMRT----------------------EGKQIAAFLVEPIQGEGGIIVPPEGYLKNAEIICRKYGVLLAV 220

PpKTFusion/1-959 DEVQSGFGRAGTFLASSQFNLQGDYYCLSKALGGGLMKIAATVIRSSHYEGEFSYIHSSTFAEDDPSCHIALSALRRLFA 398
TmFusion/1-981   DEVQTGLGRTGALFACQAEGVTPDAMTLAKALGGGLMPIGAVLCTEEVYTEEFATKHSSTFAGNTLACRAGLAALDLLTR 302
TsFusion/1-886   DEVQTGLGRTGVLFACQAEGVTPDAITLAKALGGGLMPIGAVLCTEEVYTEEFATKHSSTFAGNTLACRAGLAALDLLTR 302
TfFusion/1-902   DEIQTGLGRTGELFACDREGVEPDILLLSKALGGGLVPLGVCLSTRQAWNEEFGRLHSSTFANNNFTCAIGLAVLNKLLE 300

PpKTFusion/1-959 DDDAMLKDIRTKGAYLKASLNELKMAYPDVIADVRGRGLLVGLELHDLSV----SSSLVQASAQYNDALGYLIAGYLLQF 474
TmFusion/1-981   DDQALVRHVAETGEYLRQGLLAIQRRHPRVIREVRGRGFMLGLQFGVTRETFPG---NLLGVMGEQELLTPVIASYLLNV 379
TsFusion/1-886   DEQALVRHVAETGEYLRQGLLAIQRRHPRVIREVRGRGFMLGIQFGVDREAFPG---NLLGVMGEQELLTPVIASYLLNV 379
TfFusion/1-902   NGRQLINNAKSAGNYLLKNLEDINRQYPGVIKEIRGRGLMLGLEFNEFDG-SES---FSMKYLAEQGGFSPLLAGYLLNV 376

PpKTFusion/1-959 EALRVAPSGSNSNVIRLEPPACITLGEIDKLIAALQRVCDMLRRGDALPLAAGICADSMP--ALPARQDDFRVTE----- 547
TmFusion/1-981   EGLRVAPTLNGADVIRIEPPLIATREECDYALAAIERVVDLIDREDTAGLLRHLVGQGRVGQD--RHGATSPDHGMNGRS 457
TsFusion/1-886   EGLRVAPTLNGADVIRIEPPLIATRAECDYALAAIERVVELIDRDDTAALLRHLVSPQATTGQ--AV------------- 444
TfFusion/1-902   HKVRVAPFLNNPMTLRLQPSLTVATVEIDRALHGLERVVKALYYQDHCELYSYIIGKE--PGP--IRDFRSE-------- 444

PpKTFusion/1-959 ----------------------------------P--------------------------VENRDAKVEVVARVAFINH 567
TmFusion/1-981   AAIGRVVVGRSGTGVTRDGDGASRRATGAGELPATGRGGQVGTGTVHGGASQTPAAGSSREEPATPSDDPAEGRFAFLVH 537
TsFusion/1-886   ---------------DR---------------PAR-----------H-QPALLAVRSREPEEAVAPSGDPREGRFAFLVH 482
TfFusion/1-902   --------------------------------------------------------KKAVTGSELLPEEKPTEKFAFLIH 468

PpKTFusion/1-959 LIDADILGDVDPSLAALSPEQKRTFINRTKPE--RRAVPVGPVIIRSRLGMAVEFTLYPLCMDSDAMAEYIRSGDLDSIR 645
TmFusion/1-981   PVDLENYPEFDPGLAAFTREELADLAGRWNHL--LKPFRIGQTRVVSTCGRTAYGEFYVVPRTADELLAMP---AQEA-V 611
TsFusion/1-886   PVDLENYPEFDPSLAAFSRAELEELAGRWNHL--LKPFRIGRTRVVSAAGATAYGEFYAVPRTADEFLAMP---QAEA-V 556
TfFusion/1-902   YPSSEDVIKNNPSFKKASKDILEKLIDWEASLDAEPEVIVHLPAIKSKAGKIAEGWLIGIPYSGRHMMEMP---RKDA-V 544

PpKTFusion/1-959 EEVGRRVTDARADGCSIAGLGMYTSIVTNNCQALKIADMALTSGNALTIGMGLEAIEQGCVQQGLALCEQTAAVVGAAGN 725
TmFusion/1-981   AAVKEALDLARDGGARIVGLGAYTSVVTRGGLHLRDAGVALTTGNSFTVAAAVEAIDEATRRLGFPLAESTVAVVGATGA 691
TsFusion/1-886   AEIEEAIELARENGARIVGLGAYTSVVTRGGLHLRDAGVALTTGNSFTVVAAVEAIAEASRRLGFPLGQGTVAVVGATGA 636
TfFusion/1-902   KVLVTALDKAKALGARIVGLGAYTSVVSRGGSDLQGKGIAVTSGNSYTIATAFDALIEGARLMGIDPAESTGCVIGATGS 624

PpKTFusion/1-959 VASTYASVLSATVDHLILIGSGRDGS--VRRLEKTAQQIYADAARSILKGTAEHDRLAQRLLTLDG----FRG------- 792
TmFusion/1-981   IGRATALLLGPRVQRLLLIGNPARPEQSRRRLLRVAGEICRHVLVSAGRP--S-------PVGRAG----RVDDGDRDQE 758
TsFusion/1-886   IGRATALLLAGEVRRLVLIGNPARPEQSRRRLLRVAADLARHVLSLAASG--R--------------------------- 687
TfFusion/1-902   IGRVCAILLAEEIDKLVLVGNPEKEKTSLRRMEQLADEIYTRAFREILAYKGK-------VAKIKGIAKWLKGFLDRKRK 697

PpKTFusion/1-959 --------------------------LLHSHGQ-----KADLG--LHIARLV-DERLGANAFITVTNDL-DAIKGARIVL 837
TmFusion/1-981   NHGAPLGGSGAAFFNRFGTAEAGELAPLAAQLLDFGGWPNADAPVEEFVRRLETWMARGQCPLVITTDLDAMLPLADVVV 838
TsFusion/1-886   -----------------------PLGPLAQVLVEFGGWPDPAEPAEAFLPRLEAWLAAGRCPLVVTTDLDAMLPQADVVV 744
TfFusion/1-902   DDP--------------------EV---WNKLLTAL-ENQGFSINGYIHENLAAKGKYSNPPVKITVNIKQALLQSDLII 753

PpKTFusion/1-959 CAANAPQPFLGAEHFAERSVICDIAVPLNVHQDLPSQREDVLYMHGGIVQTPFDDGLAPNVRAYLKKGQLYACMAESVLM 917
TmFusion/1-981   TATSSTAHLVTPGNVKFGAVVCDLSRPPNVSREVRDARPDVLVIDGGVIEVPGRPS--LGWNFGFERGLVYACMAETMIL 916
TsFusion/1-886   TATSSTAHLVTPRNVKFGAVVCDLSRPPNVSREVGDARPDVLVIDGGVIEVPGRPS--MGWNFGFERGLVYACMAETMML 822
TfFusion/1-902   SASNSTNHLIGPGHLKPGSVICDVARPPDVSEAVLEQRKDVLVIEGGLVQYPDDIC--FGQNMGYEPGVNMACLSETMLL 831

PpKTFusion/1-959 GLSGMSQHGSYGD-ISREQVQQVRALAATHGFTLAQFKTQNSL----------------------------- 959
TmFusion/1-981   ALEHHYQHTSLGADLNLETILWLEELARKHGFRLAQLRSFDRPLPDEAWERLVAARSAVLGSAAR------- 981
TsFusion/1-886   ALEHHYRHTSLGADLNLETILWLKDLARQHGFRLAELRSFDRPLPAEAWERVLAARSALG-TAAR------- 886
TfFusion/1-902   ALEGTYRDFSIGLKIPVENVYYLRELAQRHGFKLATPWNKNGGVTPEVARMIKEAALKNE-NVEKIKTPKSS 902
```

Fig. S2. *Pp*KTFusion and thermophilic homolog' amino acid sequence alignment. Residues highlighted in black are identical in all sequences. Residues highlighted in grey are synonymous. Red and blue squares show the implied boundaries of the N- and C-terminal domains, respectively, with the sequence in between proposed as the linker peptide. *Pp*KTFusion N-terminal domain boundary was based on the sequence alignment at the time.

Table S6. Thermophilic fusion sequence similarity results.

Homolog	Organism	Seq. Length	Query cover	Identity
*Tm*Fusion	*Thermaerobacter marianensis* DSM 12885	981	ref	ref
*Ts*Fusion	*Thermaerobacter subterraneus* DSM 13965	886	92%	93%
*Tf*Fusion	*Thermincola ferriacetica* Z-0001	902	87%	49%

Analysis was performed using BLASTP.

Table S7. Thermophilic fusion conserved domain' general function prediction by NCBI CD-Search.

Enzyme	Conserved Domain	aa region
*Tm*Fusion ADU52204.1	ArgD acetylornithine/succinyldiaminopimelate/putrescine aminotransferase [amino acid transport and metabolism]	22-419
	COG5322 superfamily Predicted amino acid dehydrogenase [general function prediction only]	579-949
*Ts*Fusion EKP95021.1	ArgD Acetylornithine/succinyldiaminopimelate/putrescine aminotransferase [amino acid transport and metabolism]	22-419
	COG5322 superfamily Predicted amino acid dehydrogenase [General function prediction only]	468-855
*Tf*Fusion KNZ69380.1	ArgD Acetylornithine/succinyldiaminopimelate/putrescine aminotransferase [amino acid transport and metabolism]	1-418
	COG5322 superfamily Predicted amino acid dehydrogenase [General function prediction only]	513-864

Table S8. Thermophilic fusion BLASTP results summary.

Number of sequences	Query cover	Identity	Genus and Species
**<u>Tier 1 *Tm*Fusion</u>**			
10 fusion-like[a]	91-100%	71-100%	*Thermaerobacter* sp. PB12/4term, *Thermaerobacter subterraneus*, *Firmicutes bacterium*, *Symbiobacterium terraclitae*, *Symbiobacterium thermophilum* IAM 14863, *Thermaerobacter* sp. FW80
**<u>Tier 1 *Ts*Fusion</u>**			
4 fusion-like[a]	97-99%	77-100%	*Thermaerobacter* sp. PB12/4term, *Thermaerobacter marianensis*, *Thermaerobacter* sp. FW80, *Firmicutes bacterium*
**<u>Tier 1 *Tf*Fusion</u>**			
1 fusion-like[a]	100%	95-100%	*Thermincola potens*
**<u>Tier 2 *Tm*Fusion / *Ts*Fusion / *Tf*Fusion (shared)</u>**[†]			
500-600 predominantly fusion-like[a]	71-99%	28-69%	various species, predominantly from *Alkalihalobacillus*, *Firmicutes*, *Paraburkholderia*, *Pseudomonas*, *Rhodococcus*, *Streptomyces*, and *Syntrophomonadaceae* genera
**<u>Tier 3 *Tm*Fusion / *Ts*Fusion / *Tf*Fusion (shared)</u>**[†]			
400-480 predominantly TAm-like[b]	41-70%	37-67%	various species, predominantly from *Acidaminococcaceae*, *Alkaliphilus*, *Armatimonadetes*, *Clostridia*, *Firmicutes*, *Moorella*, *Pelotomaculum*, *Peptococcaceae*, *Syntrophomonadaceae*, *Thermoanaerobacter*, *Thermoanaerobacterium*, and *Thermus* genera
5-20 TAm-like[b]	24-40%	41-70%	predominantly from *Firmicutes*

[†] shared entries represent entries that were returned for all three thermophilic fusions by BLASTP

[a] entries with sequence length ranging between 650-1100 aa

[b] entries with sequence length ranging between 400-600 aa

1.1.8 Residue conservation

Protein residue conservation analysis in MSA was performed using Jensen–Shannon divergence scoring method[10] (Department of Computer Science and Lewis-Sigler Institute for Integrative Genomics, Princeton University, USA), employing an online server accessible at http://compbio.cs.princeton.edu/conservation/score.html.

Where the enzyme has not been previously characterised, conservation analysis is particularly useful for their activity and mutagenesis studies.

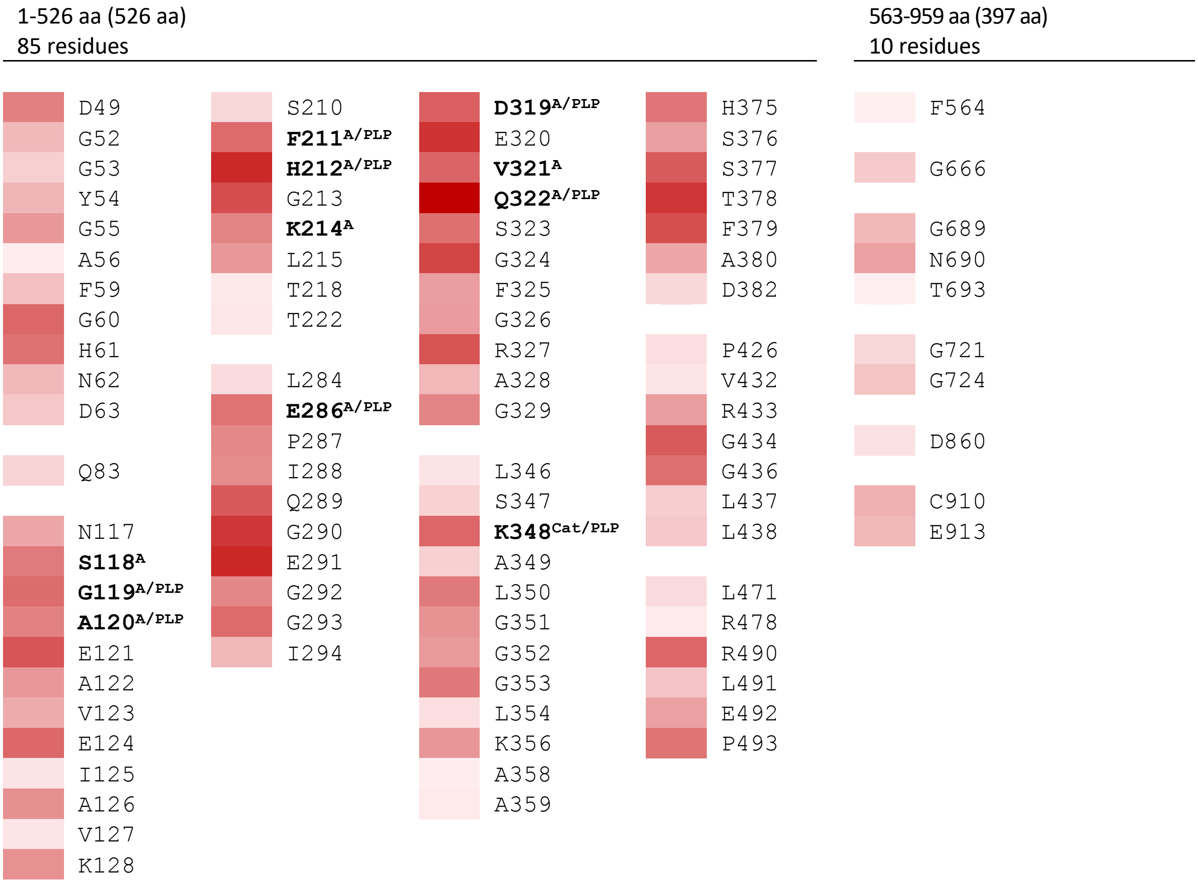

Fig. S3. *Pp*KTFusion residue conservation analysis. Heatmap of *Pp*KTFusion residues with top 10% conservation scores. A MSA, composed of a total of 359 homologs, was used as input. Darker colour indicates greater residue conservation. Residues in bold indicate predicted active site ('A') and PLP-binding regions ('PLP'), and the catalytic residue ('Cat') (in line with AAN68390.1 NCBI entry for the enzyme). Protein residue conservation analysis in MSA was performed using Jensen–Shannon Divergence scoring method,[10] employing an online server accessible at http://compbio.cs.princeton.edu/conservation/score.html. Jensen-Shannon Divergence (JSD) method determines the probability of each residue logarithmically, by evaluating sequentially neighbouring sites and comparing distribution of residues in individual sequences to that of the entire MSA.[11]

1.1.9 Structural homologs

To identify structural homologs or to predict protein structure (where those were not available), Phyre2[12] online protein fold recognition server (Structural Bioinformatics Group, Imperial College, London), accessible at http://www.sbg.bio.ic.ac.uk/phyre2/, was employed. AlphaFold[13,14] (developed by DeepMind and EMBL-EBI), accessible at https://alphafold.ebi.ac.uk, was used to generate the final putative 3D protein structures. Predicted protein 3D structures were aligned with available 3D structures, using PyMOL Molecular Graphics System (Schrödinger LLC).

Table S9. ***Pp*****KTFusion N- and C-terminal domain principal structural homologs.**

Structural Homolog	Organism	PDB ID (chain)	EC number[a]	Query aligned residues / coverage (%)	Seq. ID (%)
*Pp*KTFusion N-terminal domain: 1-526 aa (526 aa)					
class-III aminotransferase, CrmG (523 aa)	*Actinoalloteichus* sp. WH1-2216-6	5DDW(D)[b]	2.6.1	9-524 (97%)	35%
putrescine aminotransferase, YgjG-protein a-zpa963-calmodulin complex (499 aa)	*Escherichia coli*	5H7D(I)[c]	2.6.1.29, 2.6.1.82	2-519 (98%)	28%
bifunctional diaminopelargonic acid aminotransferase and dethiobiotin synthetase, DAPA AT-DTBS (831 aa)	*Arabidopsis thaliana*	4A0R(B)	2.6.1.62; 6.3.3.3	1-515 (97%)	21%
adenosylmethionine-8-amino-7-oxononanoate aminotransferase, DAPA AT (452 aa)	*Bacillus anthracis*	3N5M(D)	2.6.1.62	24-516 (93%)	23%
class-III aminotransferase (458 aa)	*Ruegeria* sp. TM1040 (formerly *Silicibacter*)	3FCR(A)	2.6.1	1-516 (97%)	21%
class-III aminotransferase (472 aa)	*Ruegeria pomeroyi* (formerly *Silicibacter*)	3HMU(A)	2.6.1	1-518 (98%)	18%
omega-transaminase, *Cv*TAm (459 aa)	*Chromobacterium violaceum*	4A6T(A)	2.6.1.18; 2.6.1.62	5-518 (97%)	19%
*Pp*KTFusion C-terminal domain: 527-959 aa (433 aa)					
long-chain acyl-[acyl-carrier-protein] reductase, AAR (341 aa)	*Synechococcus elongatus* PCC 7942 = FACHB-805 (cyanobacteria)	6JZU(A)[d]	1.2.1.80	562-953 (90%)	25%
glutamyl-tRNA reductase, GluTR (404 aa)	*Methanopyrus kandleri*	1GPJ(A)	1.2.1.70	612-853 (59%) / 658-852 (48%)	29% / 32%
glutamyl-tRNA reductase, GluTR (472 aa)	*Arabidopsis thaliana*	4N7R(B)	1.2.1.70	614-852 (59%)	22%
N-((2S)-2-amino-2-carboxyethyl)-L-glutamate dehydrogenase, SbnB (339 aa)	*Staphylococcus aureus*	4MP6(A)	1.5.1.51	655-917 (65%)	12%
glutamyl-tRNA reductase, GluTR (144 aa)	*Thermoplasma volcanium*	3OJ0(A)	1.2.1.70	664-841 (43%)	16%

[a] EC 2.6.1: transaminases; 2.6.1.18: β-alanine-pyruvate transaminase; EC 2.6.1.29: diamine transaminase; EC 2.6.1.62: adenosylmethionine-8-amino-7-oxononanoate transaminase; EC 2.6.1.82: putrescine-2-oxoglutarate transaminase; EC 6.3.3.3: dethiobiotin synthase (a cyclo-ligase – forms carbon-nitrogen bonds); EC 1.2.1.70: glutamyl-tRNA reductase (an oxidoreductase that acts on the aldehyde or oxo group using $NADP^+$); EC 1.2.1.80: long-chain / fatty acid acyl-[acyl-carrier-protein] reductase (an oxidoreductase that acts on the aldehyde or oxo group using $NAD(P)^+$); EC 1.5.1.51: N-((2S)-2-amino-2-carboxyethyl)-L-glutamate dehydrogenase (an oxidoreductase that acts on CH-NH group using NAD^+).

[b] released in August 2016 but omitted by search algorithms until 2019. [c] released in June 2017. [d] released in April 2020.

Secondary structure and fold homology were predicted with >99% confidence over the aligned regions for all homologs. Structural homologs of the *Pp*KTFusion full sequence were analogous to that returned by the *Pp*KTFusion N-terminal domain (1-526 aa). The search did not find structural homologs for the entire *Pp*KTFusion sequence, meaning that, at the time of the analysis, there were no solved structures available for such fusion enzymes in the PDB database.

Table S10. Thermophilic enzyme N-terminal domain principal structural homologs.

Structural Homolog	Organism	PDB ID (chain)	EC number[a]	Aligned residues / coverage (%)	Seq. ID (%)
N-terminal domain: *Tm*Fusion 1-435 aa (435 aa) / *Ts*Fusion 1-435 aa (435 aa) / *Tf*Fusion 1-432 aa (432 aa)					
class-III aminotransferase, CrmG (523 aa)	*Actinoalloteichus* sp. WH1-2216-6	5DDW(D)[b]	2.6.1	7-430 (97%) *Tm*TAm<br>7-430 (97%) *Ts*TAm<br>3-427 (98%) *Tf*TAm	33%<br>33%<br>33%
class-III aminotransferase, ω-TATR (451 aa)	*Thermomicrobium roseum* DSM 5159	6IO1(B)[c]	2.6.1	4-421 (95%) *Tm*TAm<br>4-421 (95%) *Ts*TAm<br>4-420 (96%) *Tf*TAm	35%<br>35%<br>33%
YgjG putrescine aminotransferase-protein a-zpa963-calmodulin complex (YgjG, 499 aa)	*Escherichia coli*	5H7D(I)[d]	2.6.1.82; 2.6.1.29	1-433 (97%) *Tm*TAm<br>1-433 (99%) *Ts*TAm<br>4-431 (98%) *Tf*TAm	38%<br>38%<br>34%
aminotransferase, PigE (861 aa)	*Serratia* sp. FS14	4PPM(B)	2.6.1	1-425 (97%) *Tm*TAm<br>1-424 (97%) *Ts*TAm<br>4-421 (96%) *Tf*TAm	41%<br>42%<br>40%
putrescine aminotransferase, YgjG (467 aa)	*Escherichia coli*	4UOX(B)	2.6.1.82; 2.6.1.29	1-423 (97%) *Tm*TAm<br>1-423 (97%) *Ts*TAm<br>4-420 (96%) *Tf*TAm	38%<br>38%<br>33%
aminoadipate semialdehyde transaminase, LysW (344 aa)	*Thermus thermophilus* HB8	1VEF(A)	2.6.1.11/2.6.1.118	1-418 (95%) *Tm*TAm<br>1-418 (95%) *Ts*TAm<br>17-415 (92%) *Tf*TAm	39%<br>38%<br>35%
acetylornithine aminotransferase, ACOAT (385 aa)	*Thermotoga maritima* MSB8	2ORD(A)	2.6.1.11	9-421 (94%) *Tm*TAm<br>2-421 (96%) *Ts*TAm<br>7-418 (95%) *Tf*TAm	37%<br>37%<br>37%
acetylornithine aminotransferase, ACOAT (376)	*Aquifex aeolicus* VF5	2EH6(A)	2.6.1.11	20-418 (91%) *Tm*TAm<br>19-418 (91%) *Ts*TAm<br>13-415 (91%) *Tf*TAm	39%<br>38%<br>37%

[a] EC 2.6.1: transaminases; 2.6.1.11: acetylornithine transaminase; 2.6.1.29: diamine transaminase; EC 2.6.1.82: putrescine-2-oxoglutarate transaminase; EC 2.6.1.118: [amino group carrier protein]-γ-(L-lysyl)-L-glutamate aminotransferase;

[b] released in August 2016 but omitted by search algorithms until 2019.

[c] released in May 2019.

[d] released in June 2017.

Table S11. Thermophilic enzyme C-terminal domain principal structural homologs.

Structural Homolog	Organism	PDB ID (chain)	EC number[a]	Aligned residues / coverage (%)	Seq. ID (%)
C-terminal domain: *Tm*Fusion 436-981 aa (546 aa)					
long-chain acyl-[acyl-carrier-protein] reductase, AAR (341 aa)	*Synechococcus elongatus* PCC 7942 = FACHB-805	6JZU(A)[b]	1.2.1.80	534-955 (76%)	27%
D-3-phosphoglycerate dehydrogenase, PGDH (528 aa)	(cyanobacteria)	1YGY(A)	1.1.1.95, 1.1.1.399	649-955 (55%)	33%
glutamyl-tRNA reductase, GluTR (404 aa)	*Mycobacterium tuberculosis*	1GPJ(A)	1.2.1.70	605-885 (51%)	34%
NADP-dependent malic enzyme (439 aa)	*Methanopyrus kandleri*	1WW8(A)	1.1.1.38	661-974 (57%)	24%
Adenosylhomocysteinase, AdoHcyase (466 aa)	*Pyrococcus horikoshii* Ot3	3N58(D)	3.3.1.1	661-980 (58%)	25%
bifunctional methylenetetrahydrofolate dehydrogenase-cyclohydrolase (281 aa)	*Brucella abortus* 2308	2C2X(B)	1.5.1.5/3.5.4.9	664-888 (40%)	45%
	Mycobacterium tuberculosis				
C-terminal domain: *Ts*Fusion 436-886 aa (451 aa)					
long-chain acyl-[acyl-carrier-protein] reductase, AAR (341 aa)	*Synechococcus elongatus* PCC 7942 = FACHB-805	6JZU(A)[b]	1.2.1.80	478-860 (76%)	28%
D-lactate dehydrogenase (329 aa)	(cyanobacteria)	4CUK(A)	1.1.1.28	561-778 (48%)	32%
phosphite dehydrogenase (336 aa)	*Salmonella enterica subsp. enterica serovar Typhi*	4E5K(C)	1.20.1.1	592-778 (41%)	38%
glycerate dehydrogenase/glyoxylate reductase (311 aa)	*Pseudomonas stutzeri*	2CUK(C)	1.1.1	593-778 (41%)	40%
C-1-tetrahydrofolate synthase (298 aa)	*Thermus thermophilus* HB8	4CJX(A)	1.5.1.5	608-793 (41%)	39%
D-lactate dehydrogenase (335 aa)	*Trypanosoma brucei brucei* TREU927	4XKJ(A)	1.1.1.28	608-793 (47%)	30%
	Sporolactobacillus inulinus CASD				
C-terminal domain: *Tf*Fusion 433-902 aa (470 aa)					
long-chain acyl-[acyl-carrier-protein] reductase, AAR (341 aa)	*Synechococcus elongatus* PCC 7942 = FACHB-805	6JZU(A)[b]	1.2.1.80	465-870 (85%)	28%
malic enzyme (487 aa)	(cyanobacteria)	3NV9(A)	1.1.1.40	594-900 (64%)	27%
NAD-dependent malate oxidoreductase (376 aa)	*Entamoeba histolytica*	1VL6(A)	1.1.1.37	594-828 (49%)	29%
D-2-hydroxyisocaproate dehydrogenase, D-HICDH (333 aa)	*Thermotoga maritima* MSB8	1DXY(A)	1.1.1	550-788 (50%)	31%
glutamyl-tRNA reductase, GluTR (404 aa)	*Lacticaseibacillus casei*	1GPJ(A)	1.2.1.70	589-800 (44%)	25%
S-adenosylhomocysteine hydrolase, AdoHcyase (404 aa)	*Methanopyrus kandleri*	3X2F(A)	3.3.1.1	597-886 (61%)	23%
	Thermotoga maritima MSB8				

[a] EC 1.1.1: oxidoreductases acting on the CH-OH groups using NAD(P)$^+$; EC 1.1.1.28: D-lactate dehydrogenase (NAD$^+$); EC 1.1.1.37: malate dehydrogenase (NAD$^+$); EC 1.1.1.38: malate dehydrogenase (oxaloacetate-decarboxylating) (NAD$^+$); EC 1.1.1.40: malate dehydrogenase (oxaloacetate-decarboxylating) (NADP$^+$); EC 1.1.1.95: phosphoglycerate dehydrogenase (NAD$^+$); EC 1.1.1.399: α-ketoglutarate reductase (NAD$^+$); EC 1.2.1.70: glutamyl-tRNA reductase (an oxidoreductase that acts on the aldehyde or oxo group using NADP$^+$); EC 1.2.1.80: long-chain / fatty acid acyl-[acyl-carrier-protein] reductase; EC 1.5.1.5: methylenetetrahydrofolate dehydrogenase (an oxidoreductase that acts on CH-NH group using NADP$^+$; EC 1.20.1.1: phosphite dehydrogenase (an oxidoreductase that acts on phosphorus or arsenic using NAD$^+$); EC 3.3.1.1: adenosylhomocysteinase (a thioether/trialkylsulfonium hydrolase – acts on ether bonds); EC 3.5.4.9: methenyltetrahydrofolate cyclohydrolase (a hydrolase that acts on non-peptide forming carbon-nitrogen bonds in cyclic amidines).

[b] released in April 2020.

1.1.10 3D Models

3D models were generated using AlphaFold (DeepMind and EMBL-EBI)[13,14] to visualise the proximity between the TAm and AAR domain active sites. *Pp*KTFusion, *Tm*Fusion, *Ts*Fusion, and *Tf*Fusion structures were comparable but highlighted differences in the linker length and slight variations in the domain organisation, (**Fig. S4**(A)). The distance between the catalytic residues in the TAm and AAR domains, which fell within the top 10% conservation scores (**Fig. S4**(B), **Fig. S3**), was determined at ca. 50 Å.

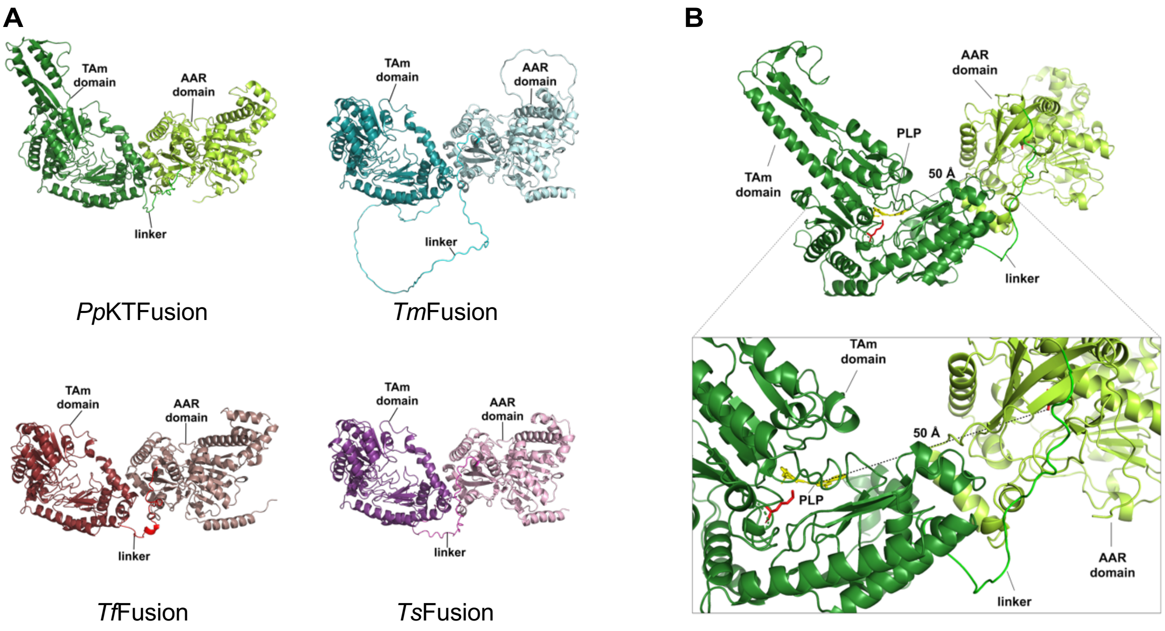

Fig. S4. 3D models of the *P. putida* KT2440 transaminase fusion and its thermophilic homologs. (A) Comparison of the predicted structures of *Pp*KTFusion (linker: 33 aa), *Tf*Fusion (linker: 29 aa), *Tm*Fusion (linker: 95 aa), and *Ts*Fusion (linker: 40 aa), highlighting the difference in the linker peptide length. Structures were generated by AlphaFold (DeepMind/EMBL-EBI), accessible at https://alphafold.ebi.ac.uk. Linker regions were predicted with very low confidence (pLDDT < 50). (B) *Pp*KTFusion monomer structure outline, showing the TAm and AAR domain active sites' proximity. The distance between the catalytic residues (C910 in the AAR domain and K348 in the TAm domain, highlighted in red) was approximately 50 Å.

1.1.11 Structural alignment

Table S12. Results of *Pp*KTFusion conserved residue mapping to AAR and thermophilic homologs through a 3D structure alignment.

C-terminal domain conserved residues				
AAR (6JZU)	*Pp*KTFusion	*Tm*Fusion	*Ts*Fusion	*Tf*Fusion
L4	F564	F534	F479	F465
G99	G666	G632	G577	G565
G130	G689	G655	G600	G588
N131	N690	N656	N601	N589
T134	T693	T659	T604	T592
G162	G721	G687	G632	G620
G165	G724	G690	G635	G623
D244	D860	D861	D767	D776
C294	C910	C909	C815	C824
E297	E913	E912	E818	E827
^{162}GATGDIG^{168}	^{721}GAAGNVA^{727}	^{687}GATGAIG^{693}	^{632}GATGAIG^{638}	^{620}GATGSIG^{626}

Residue C910 was among residues with the top 10% conservation scores in *Pp*KTFusion (**Fig. S3**). Mapping primary sequence conserved residues from the *Pp*KTFusion C-terminal domain back onto the AAR sequence showed that 9 out of 10 residues were fully conserved, despite the low (25%-28%) primary sequence similarity, (**Table S12**).

1.2 Protein expression and purification

1.2.1 Protein expression – general protocol

E. coli BL21(DE3) and Rosetta™ 2(DE3) glycerol stocks (50 µL) containing plasmids with target genes were inoculated in 5-10 mL of LB or TB in 50 mL Falcon tubes supplemented with an appropriate antibiotic (kanamycin at 50 µg.mL^{-1} final concentration for pET-28a(+) plasmids in *E. coli* BL21(DE3); or kanamycin at 50 µg.mL^{-1} and chloramphenicol at 25 µg.mL^{-1} final concentration to maintain pET-28a(+) and the host's pRARE2 plasmid, in Rosetta™ 2(DE3)). Cultures were incubated in a shaking incubator at 37 °C, 250 rpm, overnight. The following day, these starter cultures were used to inoculate 100-400 mL of LB or TB in 2 L baffled conical flasks at 1-2% v.v^{-1}, adding antibiotic as stated and incubating at 30-37 °C, 180-230 rpm, until OD_{600} 0.4-0.8 (measured by Jenway 7315 spectrophotometer in 1 mL aliquots). Cultures were induced with 1 mM IPTG and incubated for 5-72 hours at 18-37 °C, 180-230 rpm, for protein expression.

Thirty minutes prior to harvesting, cultures were supplemented with a co-factor at 0.1-0.5 mM final concentration (PLP for *Cv*TAm, *Pp*KTTAm, *Tm*TAm, *Ts*TAm, *Tf*TAm; PLP and NAD^+/$NADP^+$ for *Pp*KTFusion, *Tm*Fusion, *Ts*Fusion, *Tf*Fusion; and NAD^+/$NADP^+$ for *Pp*KTDHR, *Tm*DHR, *Ts*DHR, *Tf*DHR. A small amount of culture was removed for whole cell SDS-PAGE analysis. Then, cultures were pelleted by centrifugation at 10,000 *x g* for 10 mins at 4 °C in an Eppendorf centrifuge 5810R or Beckman Coulter Avanti J-20 XPI Centrifuge. Pellets were either used immediately or stored at -80 °C until needed. Protein size was estimated by ExPASy ProtParam online tool. Expression details concerning individual enzyme sets are provided in Figures.

1.2.2 Lysate preparation by sonication

Cell pellets were resuspended in IMAC1 buffer (20 mM NaPi, 0.5 M NaCl, 5 mM imidazole pH 7.4), for protein purification at 1:25 ratio of buffer to culture volume, adding appropriate co-factors to 0.1 mM and mixing to homogeneity on ice. The cell suspension was sonicated on ice in 7.5 mL batches 10 s ON 10 s OFF for ~20 cycles, or until the suspension became homogeneous, at 8-micron amplitude using MSE Sanyo Soniprep150. Lysed cells were spun down at 10,000 x *g* for 30-45 mins at 4 °C, after which the supernatant, or soluble protein fraction, was immediately transferred to new tubes. Both soluble (supernatant) and insoluble (pelleted debris) fractions were used for SDS-PAGE analysis. The clarified cell lysate was used for protein purification.

1.2.3 Enzyme purification

The clarified cell lysate was loaded onto a 10 mL column containing 7.5 mL Ni Sepharose® 6 Fast Flow (Cytiva), for His-tag gravity-flow purification. The column was washed with 1 x cv of IMAC1 (20 mM NaPi, 0.5 M NaCl, 5 mM imidazole, pH 7.4), followed by 5 x cv of IMAC2 (20 mM NaPi, 0.5 M NaCl, 20 mM imidazole, pH 7.4), 5 x cv IMAC3 (20 mM NaPi, 0.5 M NaCl, 50 mM imidazole, pH 7.4), and 2 x cv IMAC4 (20 mM NaPi, 0.5 M NaCl, 100 mM imidazole, pH 7.4). The protein was eluted with 1-2 x cv of IMAC5 (20 mM NaPi, 0.5 M NaCl, 500 mM imidazole, pH 7.4). A VM20 Vacuum Manifold (Sigma-Aldrich), attached to a vacuum tap, was used to speed up the washing steps (IMAC1-IMAC4), utilizing vacant butterfly valves for regulation of the suction speed. Collection of the elution fraction was initiated once protein presence was confirmed by the Bradford reagent (detected by mixing 5 µL from the eluting drops with 250 µL of the reagent). The purified enzyme was supplemented with a co-factor(s) and precipitated using ammonium sulfate to 70-80% saturation, then stored at 4 °C until needed.

The observed molecular weight of the truncated thermophilic TAms was somewhat lower than predicted by the ProtParam tool, presumably due to a high content of non-polar residues[15] (58% in *Tm*TAm, 58% in *Ts*TAm, and 54% in *Tf*TAm), (**Fig. S6, Fig. S7**, **Fig. S8**, and **Fig. S18**, **Fig. S21**, and **Fig. S24**).

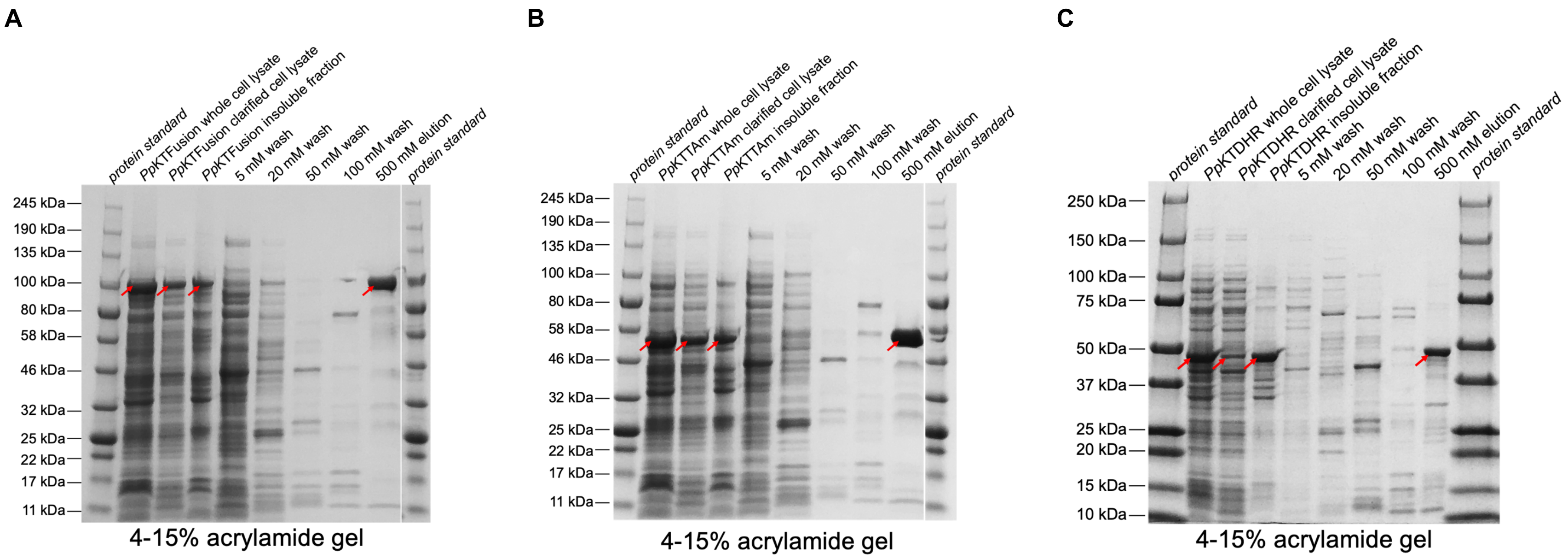

Fig. S5. SDS-PAGE of *Pp*KTFusion, *Pp*KTTAm, and *Pp*KTDHR expression, post-optimisation. Denaturing polyacrylamide gels of (A) *Pp*KTFusion (107 kDa, 979 aa), (B) *Pp*KTTAm (60 kDa, 546 aa), and (C) *Pp*KTDHR (49 kDa, 454 aa) showing the respective enzyme bands in whole cells, clarified cell lysate, insoluble fraction, and the purification steps (indicated by red arrows). All ORFs contained a N-terminal His_6-tag and were expressed in *E. coli* BL21(DE3), inducing with 1 mM IPTG at 0.5-0.6 OD_{600} (*Pp*KTFusion and *Pp*KTTAm) or 0.8-1.0 OD_{600} (*Pp*KTDHR), and incubating at 18 °C for 72 hours at 180 rpm. PLP and $NAD(P)^{+}$[a] (at 0.5 mM) co-factors were added 30 minutes prior to cell harvesting. Lysates were prepared using sonication, purifying enzymes from the soluble fraction via a nickel column. Protein size was estimated by ExPASy ProtParam online tool. *Pp*KTFusion – fusion; *Pp*KTTAm – N-terminal domain; *Pp*KTDHR – C-terminal domain. [a] PLP and $NAD(P)^+$ for *Pp*KTFusion, PLP for *Pp*KTTAm; $NAD(P)^+$ for *Pp*KTDHR.

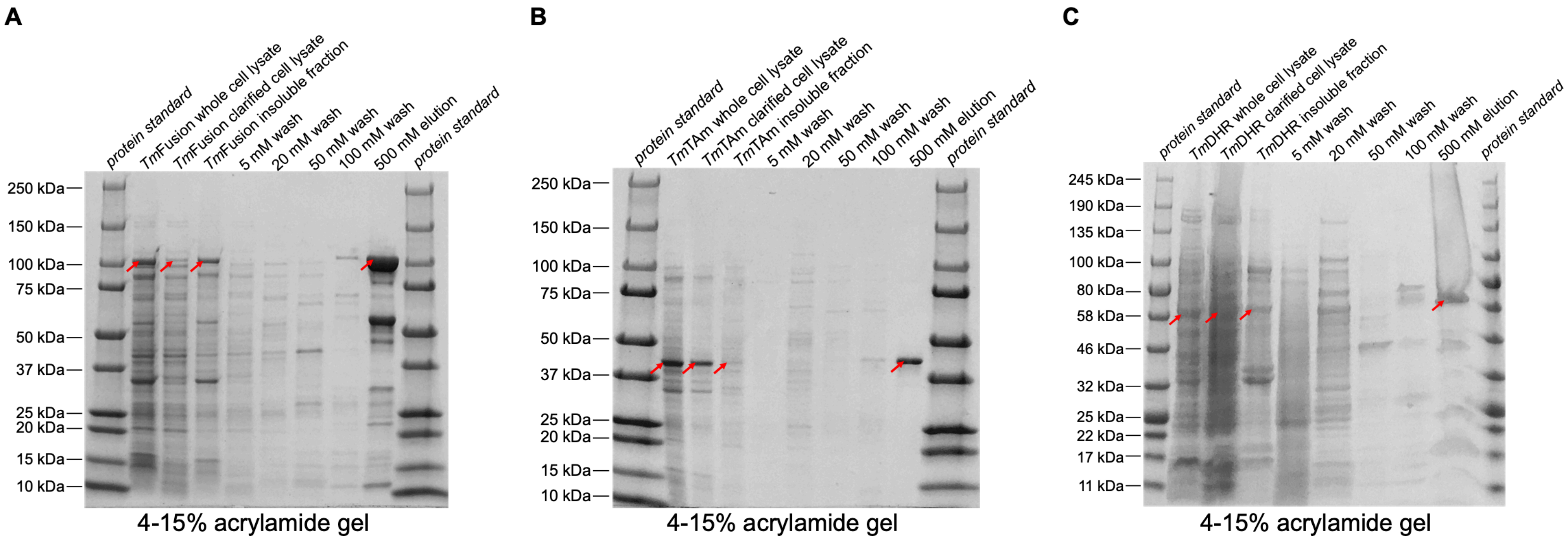

Fig. S6. SDS-PAGE gel of *Tm*Fusion, *Tm*TAm, and *Tm*DHR expression. Denaturing polyacrylamide gels of (A) *Tm*Fusion (107 kDa, 1001 aa), (B) *Tm*TAm (~43 kDa (observed), 50 kDa (predicted), 455 aa), and (C) *Tm*DHR (60 kDa, 567 aa) showing the respective enzyme bands in whole cells, clarified cell lysate, insoluble fraction, and the purification steps (indicated by red arrows). *Tm*Fusion and *Tm*TAm were expressed in *E. coli* BL21(DE3), *Tm*DHR was expressed in *E. coli* Rosetta™ 2(DE3), inducing with 1 mM IPTG at 0.5-0.6 OD_{600} (*Tm*Fusion and *Tm*TAm) or 0.8-0.9 OD_{600} (*Tm*DHR), and incubating at 30/37 °C for 72 hours at 180 rpm. Sorbitol (0.5 M)[16] was added to *Tm*Fusion culture at scale up. PLP and NAD(P)$^{+a}$ (at 0.1 mM) co-factors were added 30 minutes prior to cell harvesting. Lysates were prepared using sonication, purifying enzymes from the soluble fraction via a nickel column. Protein size for *Tm*Fusion, *Tm*TAm and *Tm*DHR was estimated by ExPASy ProtParam online tool. [a] PLP and NAD(P)$^+$ for *Tm*Fusion, PLP for *Tm*TAm; NAD(P)$^+$ for *Tm*DHR.

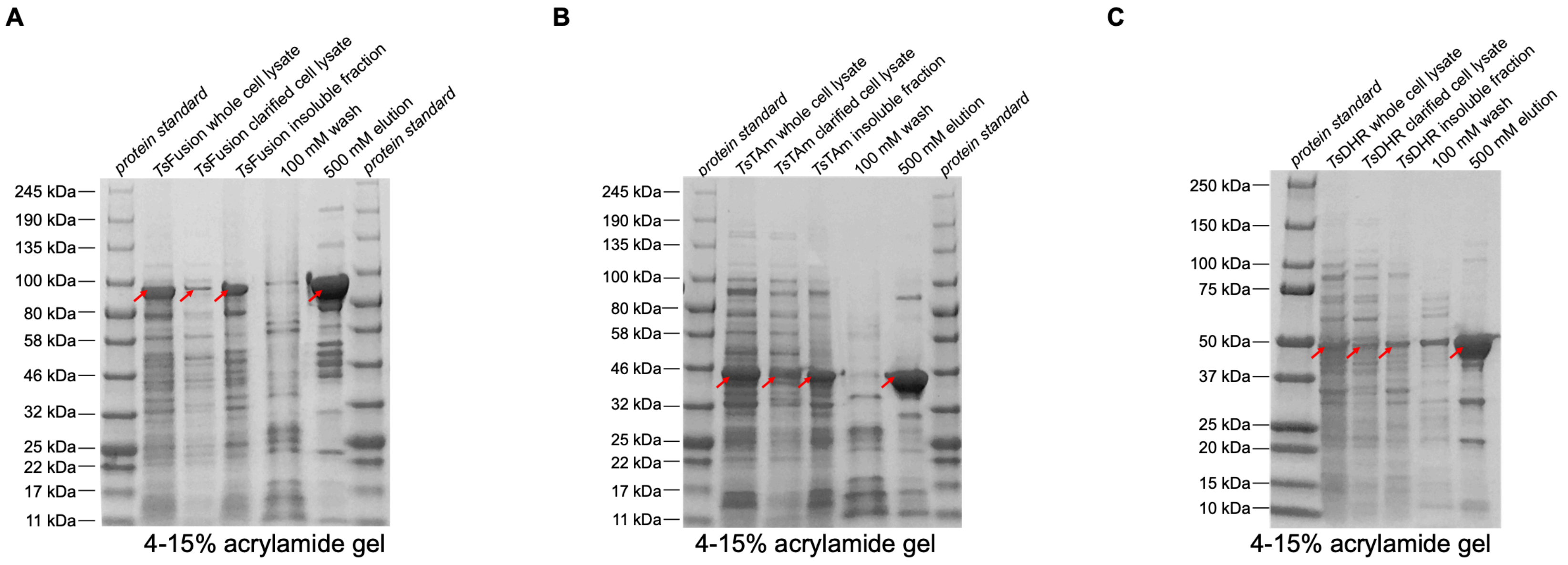

Fig. S7. SDS-PAGE gel of *Ts*Fusion, *Ts*TAm, and *Ts*DHR expression. Denaturing polyacrylamide gels of (A) *Ts*Fusion (98 kDa, 906 aa), (B) *Ts*TAm (~43 kDa (observed), 50 kDa (predicted), 455 aa), and (C) *Ts*DHR (51 kDa, 472 aa) showing the respective enzyme bands in whole cells, clarified cell lysate, insoluble fraction, and the purification steps (indicated by red arrows). *Ts*Fusion and *Ts*TAm were expressed in *E. coli* Rosetta™ 2(DE3), *Ts*DHR was expressed in *E. coli* BL21(DE3), inducing with 1 mM IPTG at 0.5-0.6 OD_{600} (*Ts*Fusion, *Ts*TAm, and *Ts*DHR) and incubating at 37 °C for 72 hours at 180 rpm. Sorbitol (0.5 M)[16] was added to *Ts*Fusion and *Ts*TAm cultures at scale up. PLP and $NAD(P)^{+}$[a] (at 0.1 mM) co-factors were added 30 minutes prior to cell harvesting. Lysates were prepared using sonication, purifying enzymes from the soluble fraction via a nickel column. Protein size for *Ts*Fusion, *Ts*TAm and *Ts*DHR was estimated by ExPASy ProtParam online tool. [a] PLP and $NAD(P)^{+}$ for *Ts*Fusion, PLP for *Ts*TAm; $NAD(P)^{+}$ for *Ts*DHR.

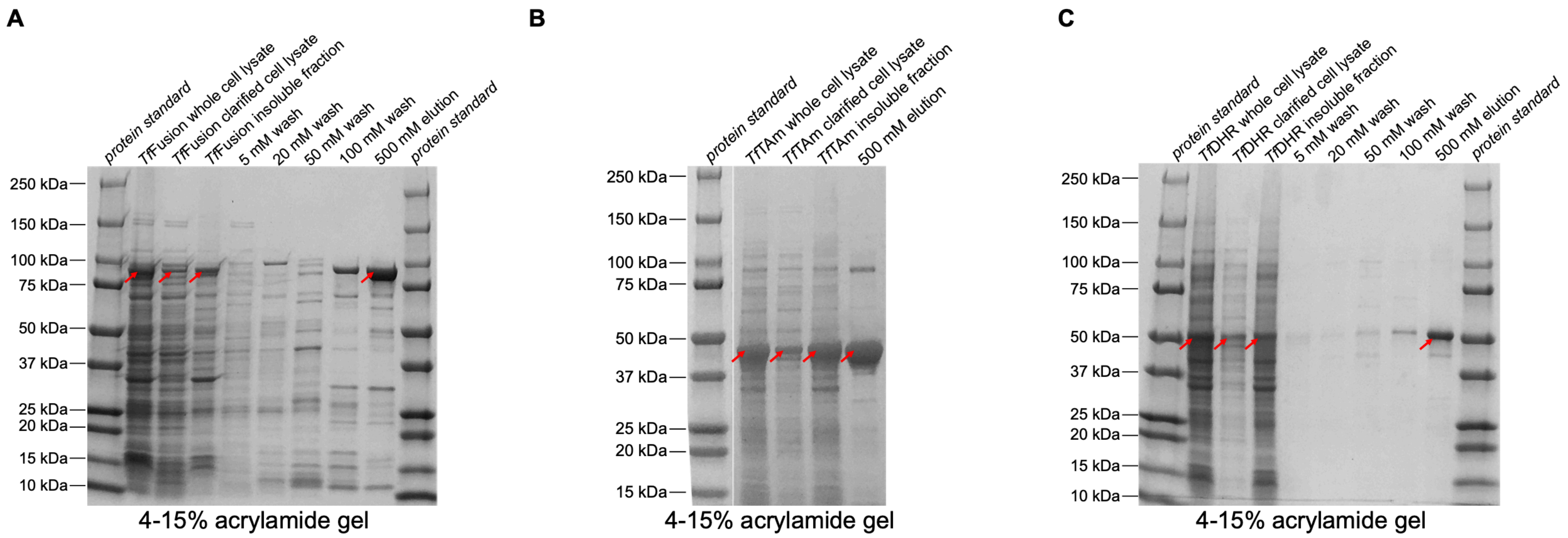

Fig. S8. SDS-PAGE gel of *Tf*Fusion, *Tf*TAm, and *Tf*DHR expression. Denaturing polyacrylamide gels of (A) *Tf*Fusion (101 kDa, 922 aa), (B) *Tf*TAm (~43 kDa (observed), 50 kDa (predicted), 452 aa), and (C) *Tf*DHR (54 kDa, 491 aa) showing the respective enzyme bands in whole cells, clarified cell lysate, insoluble fraction, and the purification steps (indicated by red arrows). *Tf*Fusion and *Tf*TAm were expressed in *E. coli* Rosetta™ 2(DE3), *Tf*DHR was expressed in *E. coli* BL21(DE3), inducing with 1 mM IPTG at 0.5-0.6 OD_{600} (*Tf*Fusion, *Tf*TAm, and *Tf*DHR) and incubating at 37 °C for 72 hours at 180 rpm. Sorbitol (0.5 M)[16] was added to *Tf*Fusion and *Tf*TAm cultures at scale up. PLP and $NAD(P)^{+a}$ (at 0.1 mM) co-factors were added 30 minutes prior to cell harvesting. PLP and $NAD(P)^{+a}$ (at 0.1 mM) co-factors were added 30 minutes prior to cell harvesting. Lysates were prepared using sonication, purifying enzymes from the soluble fraction via a nickel column. Protein size for *Tf*Fusion, *Tf*TAm and *Tf*DHR was estimated by ExPASy ProtParam online tool. [a] PLP and $NAD(P)^+$ for *Tf*Fusion, PLP for *Tf*TAm; $NAD(P)^+$ for *Tf*DHR.

1.3 Enzyme assays

1.3.1 Colorimetric assays with purified enzymes

Qualitative screening of transaminase activity was carried out via colorimetric assays using Ni-column-purified enzymes. Reactions were performed at 200 µL in 96-well plates at 30 °C (*Pp*KTFusion, *Pp*KTTAm, *Cv*TAm) or at 50 °C (*Tm*Fusion, *Ts*Fusion, *Tf*Fusion, *Tm*TAm, *Ts*TAm, *Tf*TAm) for 18 hours. Reactions contained 50 mM HEPES buffer at pH 7.5, 25 mM 2-(4-nitrophenyl)ethan-1-amine hydrochloride **44** as amine donor, 10 mM amino acceptor / substrate (keto acids: pyruvate **3**, α-ketoglutarate **4**, HPA **5**, 2-ketobutyrate **17**, 4-hydroxyphenylpyruvate **18**, 4-methyl-2-oxovalerate **19**, levulinate **20**, oxaloacetate **21**; aldehydes: benzaldehyde **1**, hexanal **2**, 2-phenylacetaldehyde **12**, 3-phenylproprionaldehyde **13**, butanal **14**, trans cinnamaldehyde **15**, vanillin **16**; ketones: ethyl acetoacetate **6**, acetophenone **7**, 1-indanone **8**, 2-heptanone **9**, 1,2-cyclohexanedione **10**, ethyl 4-oxocyclohexanecarboxylate **11**, 4-phenylbutanone **22**, cyclohexanone **23**, 3-acetyl-1-propanol **24**), 1.0 mM PLP, DMSO at 0-10% $v.v^{-1}$, and purified enzyme at 0.01 $mg.mL^{-1}$. Reactions were initiated by the addition of enzyme and performed in either duplicate or triplicate. Control reactions were prepared as above but lacked an enzyme (C1). All chemical stocks were prepared at 10X concentration in 50% or 100% DMSO (except **3, 21** and **44**, which were prepared in 100% dH_2O), to ensure solubility, resulting in 0%, 5% or 10% $v.v^{-1}$ DMSO final reaction concentration.

1.3.2 End-point assays

Transaminase activity with various amine donors was determined by analysis of the conversion of 3-phenylpropionaldehyde **13** to 3-phenyl-1-propylamine **43**, using HPLC. Reactions were performed at 200 µL in 96-well plates at 30 °C (*Pp*KTFusion, *Pp*KTTAm, *Cv*TAm) or at 50 °C (*Tm*Fusion, *Ts*Fusion, *Tf*Fusion, *Tm*TAm, *Ts*TAm, *Tf*TAm) for 18 hours. Reactions contained 50 mM HEPES buffer at pH 7.5, 25 mM amine donor (1-aminoindan **25**, 2-aminoheptane **26**, 2-phenylethylamine **27**, 4-phenylbutan-2-amine **28**, 6-aminohexanoic acid **29**, benzhydrylamine **30**, benzylamine **31,** cyclohexanamine **32**, D-alanine **33**, ethyl 3-aminobutyrate **34**, L-alanine **35**, L-glutamate **36**, L-serine **37**, (*S*)-MBA **38**, L-lysine **39**, L-arginine **40**, L-aspartate **41**, L-glutamine **42**), 10 mM amine acceptor / substrate (**13**)**,** 1.0 mM PLP, DMSO at 10-20% $v.v^{-1}$, and purified enzyme at 0.05 $mg.mL^{-1}$. Reactions were initiated by the addition of enzyme, terminated by freezing at -80 °C; and performed in duplicate. Control reactions were prepared as above but lacked an enzyme (C1) – these were subtracted from enzyme assays to remove any background signals. All amine donor stocks were prepared at 10X concentration in 50% or 100% DMSO (except **33**, **35**, **36**, **37**, **39**, **40**, **41**, and **42**, which were prepared in 100% dH_2O). Amine acceptor 3-phenylpropionaldehyde **13** was prepared at 10X in 100% DMSO, resulting in 10% or 20% $v.v^{-1}$ DMSO final reaction concentration. Product yield in samples was determined using the 3-phenyl-1-propylamine calibration curve (**Fig. S9)**. Errors are reported as standard deviations.

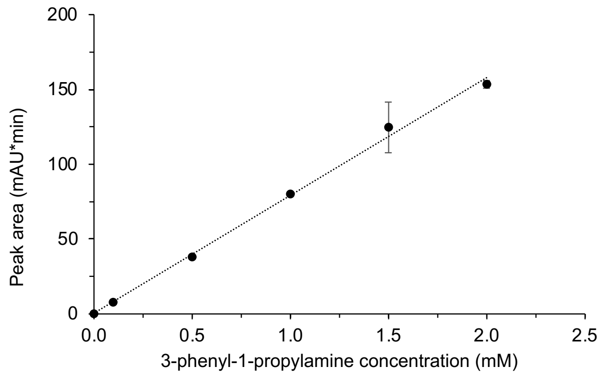

Fig. S9. Typical 3-phenyl-1-propylamine HPLC standard curve used to determine product concentration in samples. y = 79.043x; R^2 = 0.9986. Experiments were performed in duplicate. SD <2.6, except measurements at 1.5 mM, where SD was 17.

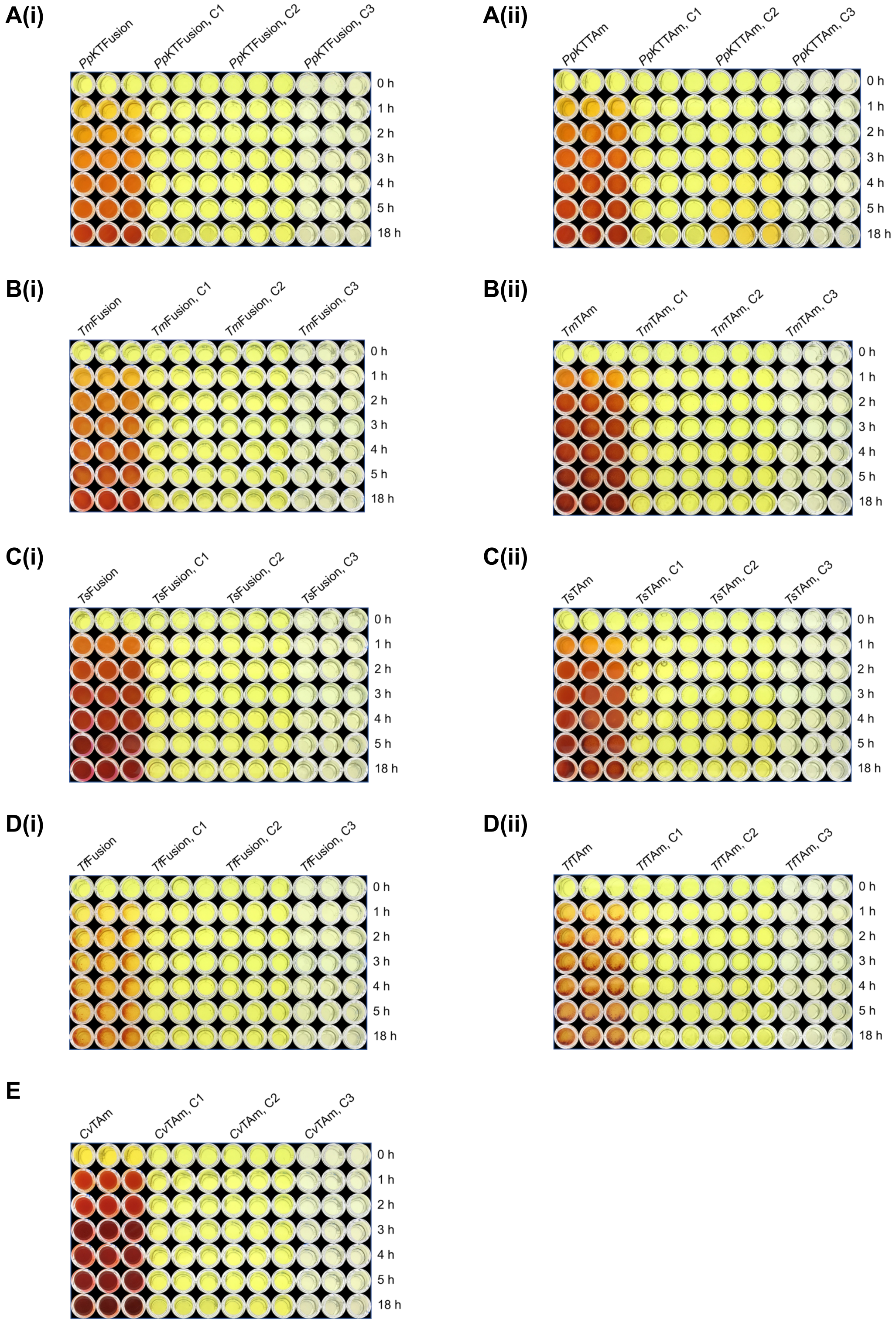

Fig. S10. Colour intensity development in control samples. *Pp*KTFusion (A(i))/*Pp*KTTAm (A(ii)), *Tm*Fusion (B(i))/*Tm*TAm (B(ii)), *Ts*Fusion (C(i))/*Ts*TAm (C(ii)), and *Tf*Fusion (D(i))/*Tf*TAm (D(ii)), and *Cv*TAm (E). Assays contained 50 mM HEPES buffer at pH 7.5, 25 mM 2-(4-nitrophenyl)ethan-1-amine **44** as amine donor, 10 mM substrate pyruvate **3**, and purified enzyme at 0.01 mg.mL^{-1}. Assays were performed in triplicate and run alongside no enzyme C1, no substrate C2, and no amine donor C3 controls. Reactions were initiated by the addition of enzyme and run at 30 °C (*Pp*KTFusion, *Pp*KTTAm, *Cv*TAm) or at 50 °C (*Tm*Fusion, *Tm*TAm, *Ts*Fusion, *Ts*TAm, *Tf*Fusion, and *Tf*TAm) for 18 hours. The dark red colouration in samples indicates conversion ≥20%.

1.3.3 Thermostability assays

Transaminase activity was determined by analysing the conversion of 3-phenylpropionaldehyde **13** to 3-phenyl-1-propylamine **43**, employing L-glutamate **36** as an amine donor, using HPLC. Reactions were performed in 200 µL volumes in 96-well plates at 50 °C for 2 hours. Each reaction contained 50 mM HEPES buffer at pH 7.5, 175 mM L-glutamate **36**, 52.5 mM 3-phenylpropionaldehyde **13**, 0.1 mM PLP, 0.1 mM $NADP^+$, 10.5% v.v^{-1} DMSO, and purified enzyme *Tf*Fusion at 0.1 mg.mL^{-1}. To measure the enzyme's residual activity (A_{Res}) following heat treatment, the enzyme was incubated with cofactors at 60 °C and 70 °C for 30, 60 and 120 minutes, then cooled on ice for 2 hours prior to initiating reactions. The enzyme's initial activity (A_0) was measured using untreated enzyme. To run assays, all reaction solutions, except for the enzyme, were equilibrated to 50 °C. Reactions were initiated by the addition of the enzyme and terminated by freezing at -80 °C; and performed in triplicate. Control reactions, prepared as above but without enzyme, were subtracted from enzyme assays to remove background signals. The amine donor stock was prepared at 1 M in 100% dH_2O. The amine acceptor, 3-phenylpropionaldehyde **13**, was prepared at 500 mM in 100% DMSO, resulting in 10.5% DMSO final reaction concentration. Product yield in samples was determined using a 3-phenyl-1-propylamine calibration curve. Errors are reported as standard deviations, (**Fig. S11**).

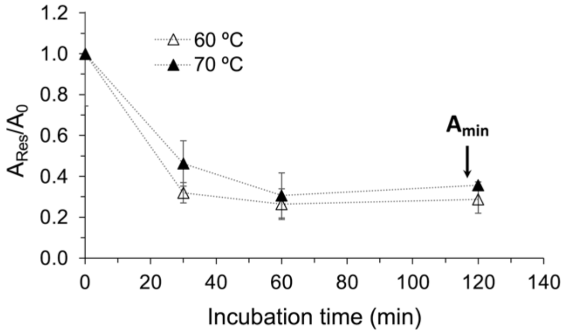

Fig. S11. Thermostability analysis of *Tf*Fusion. The graph is showing a decrease in the ratio of residual activity (A_{res}) relative to the initial activity (A_0) following incubation at 60 °C and 70 °C over various time periods.

1.4 Analytical HPLC

Analytical HPLC was performed using a DionexTM UltiMateTM 3000 HPLC System, a DionexTM UltiMateTM 3000 Autosampler, a DionexTMUltiMateTM 3000 RS Pump, a UltiMateTM 3000 RS Diode Array Detector, and a DionexTM UltiMateTM 3000 Column Compartment (ThermoFisher Scientific). Reverse-phase chromatography method was used for quantitative achiral analysis of end-point assay reaction components. Compound separation was achieved employing two mobile phases – (a) dH_2O with 0.1% v.v^{-1} TFA, and (b) acetonitrile – with ACE 5 C18 column (150 × 4.6 mm) (**Fig. S12),** at 1.0 mL.min^{-1} flow rate. The injection volume was 20 µL, with column oven at 30 °C and sample chamber at 4 °C. Standards (see **Fig. S9**) were run to determine and validate compound concentrations and retention times. A 20-minute method, with UV absorbance at 210 nm, was used with all transaminase assays.

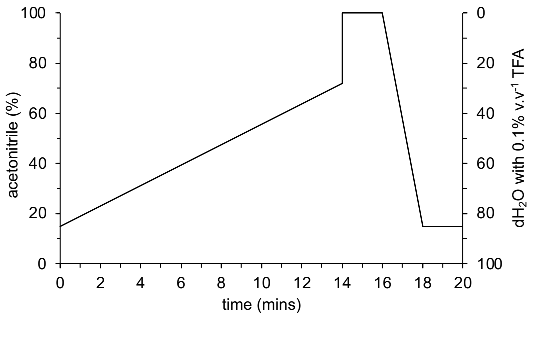

Fig. S12. Analytical HPLC method.

1.5 Kinetic parameters of *Pp*KTFusion/*Pp*KTTAm and *Tf*Fusion/*Tf*TAm

Kinetic parameters were calculated by fitting data to Michaelis-Menten or substrate inhibition models using non-linear regression (least-squares) analysis in Prism 9 (GraphPad Software LLC). For both models, the confidence interval for the interpolation of the unknowns from a standard curve was set to 95%. For the Michaelis-Menten model, to achieve the best fit, the convergence criteria was set to strict, selecting 1/Y^2 weighting method. For the substrate inhibition model, to achieve the best fit, the convergence criteria was set to medium (default), and no weighting was applied (default). For both models, the mean Y values of each point was used, (**Fig. S13**).

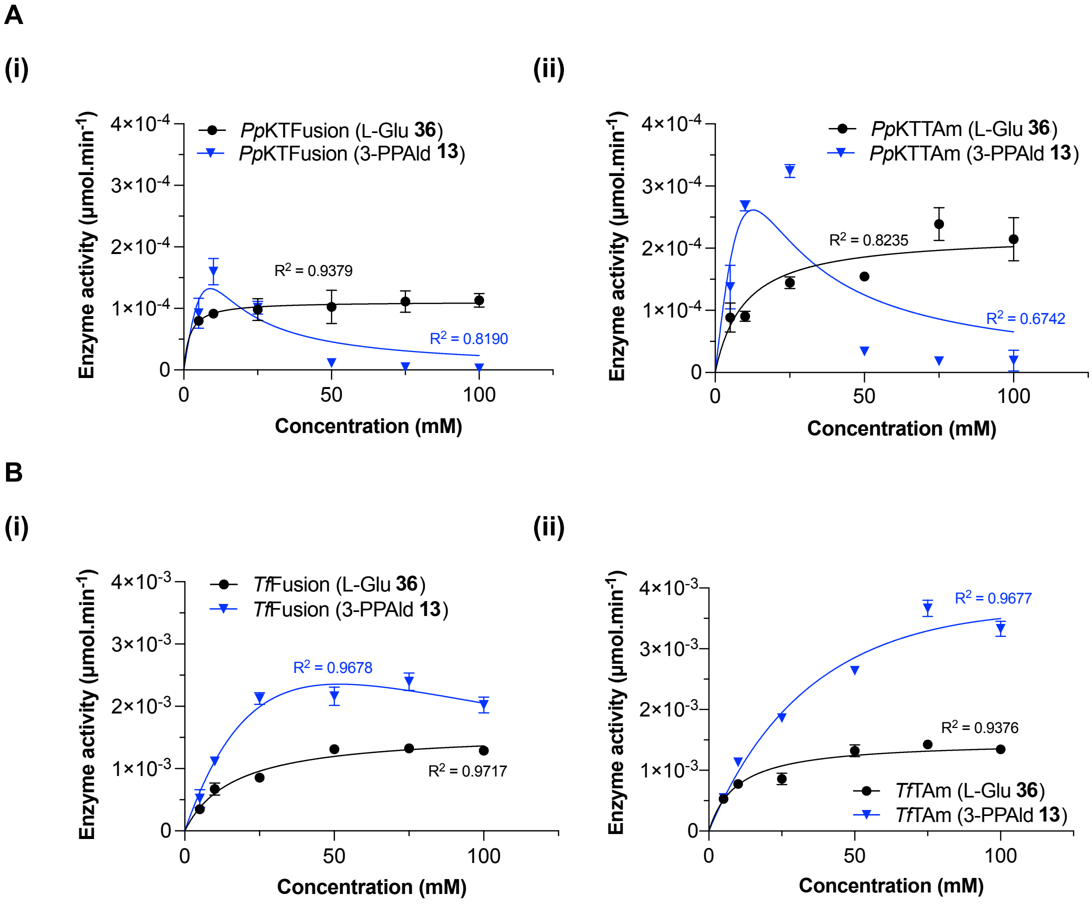

Fig. S13. Non-linear regression plots of *Pp*KTFusion/*Pp*KTTAm and *Tf*Fusion/*Tf*TAm. The L-glutamate data was best fitted to the Michaelis-Menten model ($v = \frac{V_{max} \times [S]}{K_m + [S]}$) and the 3-phenylpropionaldehyde data was best fitted to the substrate inhibition model ($v = \frac{V_{max} \times [S]}{K_m + [S] \times (1 + \frac{[S]}{K_i})}$) by the non-linear regression analysis. Where values are large enough, SD is shown as error bars. Analysis and curve fitting were performed by GraphPad Prism 9.

2. Protein sequences

2.1 Pseudomonas putida KT2440 PP_2782

2.1.1 *Pp*KTFusion protein sequence

MGSSHHHHHHSSGLVPRGSH**M**TVMDYRDFVRPKFVGLLQALGLECEFERALGSQLFYRNPKGDMVTVTDFLGGYGAALFGH
NDPQFVDQLCALLRSDVPFNAQMSIRGAAGQLGRALSDAFNRELKNTERYISTFSNSGAEAVEIAVKHAEFRRQKSLQKQF
DDIDFTLASLTASEHAYRELDVADLDLPAGVLPATLNSVTLRQVVEAVRQHNLAQLHIEPVFVALRGSFHGKLVNTVQLTY
GRQYRAPFARFGLNVEFIDPQQPHQLQELPARHTHHWLSLQWDGEHLHVLQLPFSAITAVLMEPIQGEGGINEFAAEFYLG
LRKLCNEQQCPLVVDEVQSGFGRAGTFLASSQFNLQGDYYCLSKALGGGLMKIAATVIRSSHYEGEFSYIHSSTFAEDDPS
CHIALSALRRLFADDDAMLKDIRTKGAYLKASLNELKMAYPDVIADVRGRGLLVGLELHDLSVSSSLVQASAQYNDALGYL
IAGYLLQFEALRVAPSGSNSNVIRLEPPACITLGEIDKLIAALQRVCDMLRRGDALPLAAGICADSMPALPARQDDFRVTE
PVENRDAKVEVVARVAFINHLIDADILGDVDPSLAALSPEQKRTFINRTKPERRAVPVGPVIIRSRLGMAVEFTLYPLCMD
SDAMAEYIRSGDLDSIREEVGRRVTDARADGCSIAGLGMYTSIVTNNCQALKIADMALTSGNALTIGMGLEAIEQGCVQQG
LALCEQTAAVVGAAGNVASTYASVLSATVDHLILIGSGRDGSVRRLEKTAQQIYADAARSILKGTAEHDRLAQRLLTLDGF
RGLLHSHGQKADLGLHIARLVDERLGANAFITVTNDLDAIKGARIVLCAANAPQPFLGAEHFAERSVICDIAVPLNVHQDL
PSQREDVLYMHGGIVQTPFDDGLAPNVRAYLKKGQLYACMAESVLMGLSGMSQHGSYGDISREQVQQVRALAATHGFTLAQ
FKTQNSL

Fig. S14. *Pp*KTFusion protein sequence. The protein sequence of *Pp*KTFusion with a N-terminal His_6-tag**.** The start methionine (M) residue is shown in bold. The N-terminal His_6-tag is highlighted in green; the thrombin peptide sequence is highlighted in turquoise. The proposed linker region is highlighted in red.

2.1.2 *Pp*KTTAm protein sequence

MGSSHHHHHHSSGLVPRGSH**M**TVMDYRDFVRPKFVGLLQALGLECEFERALGSQLFYRNPKGDMVTVTDFLGGYGAALFGH
NDPQFVDQLCALLRSDVPFNAQMSIRGAAGQLGRALSDAFNRELKNTERYISTFSNSGAEAVEIAVKHAEFRRQKSLQKQF
DDIDFTLASLTASEHAYRELDVADLDLPAGVLPATLNSVTLRQVVEAVRQHNLAQLHIEPVFVALRGSFHGKLVNTVQLTY
GRQYRAPFARFGLNVEFIDPQQPHQLQELPARHTHHWLSLQWDGEHLHVLQLPFSAITAVLMEPIQGEGGINEFAAEFYLG
LRKLCNEQQCPLVVDEVQSGFGRAGTFLASSQFNLQGDYYCLSKALGGGLMKIAATVIRSSHYEGEFSYIHSSTFAEDDPS
CHIALSALRRLFADDDAMLKDIRTKGAYLKASLNELKMAYPDVIADVRGRGLLVGLELHDLSVSSSLVQASAQYNDALGYL
IAGYLLQFEALRVAPSGSNSNVIRLEPPACITLGEIDKLIAALQRVCDMLRRGDALPLAA

Fig. S15. *Pp*KTTAm protein sequence. The protein sequence of the *Pp*KTFusion N-terminal domain (1-526 aa) (*Pp*KTTAm) with a N-terminal His_6-tag. The start methionine (M) residue is shown in bold. The N-terminal His_6-tag is highlighted in green; the thrombin peptide sequence is highlighted in turquoise.

2.1.3 *Pp*KTDHR protein sequence

MGSSHHHHHHSSGLVPRGSH**M**GICADSMPALPARQDDFRVTEPVENRDAKVEVVARVAFINHLIDADILGDVDPSLAALSP
EQKRTFINRTKPERRAVPVGPVIIRSRLGMAVEFTLYPLCMDSDAMAEYIRSGDLDSIREEVGRRVTDARADGCSIAGLGM
YTSIVTNNCQALKIADMALTSGNALTIGMGLEAIEQGCVQQGLALCEQTAAVVGAAGNVASTYASVLSATVDHLILIGSGR
DGSVRRLEKTAQQIYADAARSILKGTAEHDRLAQRLLTLDGFRGLLHSHGQKADLGLHIARLVDERLGANAFITVTNDLDA
IKGARIVLCAANAPQPFLGAEHFAERSVICDIAVPLNVHQDLPSQREDVLYMHGGIVQTPFDDGLAPNVRAYLKKGQLYAC
MAESVLMGLSGMSQHGSYGDISREQVQQVRALAATHGFTLAQFKTQNSL

Fig. S16. *Pp*KTDHR protein sequence. The protein sequence of the *Pp*KTFusion C-terminal domain (527-959 aa) (*Pp*KTDHR) with a N-terminal His_6-tag. The start methionine (M) residue is shown in bold. The N-terminal His_6-tag is highlighted in green; the thrombin peptide sequence is highlighted in turquoise. The proposed linker region is highlighted in red.

2.2 Thermaerobacter marianensis DSM 12885 Tmar_2123

2.2.1 *Tm*Fusion protein sequence

MGSSHHHHHHSSGLVPRGSH**M**HPFRQYVNPHLGELLEQIQMDKRFVRGQGCWLWDEDGRRYLDFVAAYGALPFGFNPPEIW
AALEEARRQGEPSFIQPSFLNAAGELARRLIEVAPPGLRYVTFANSGAEAVEAAIKLARAATGRPRILSTENAFHGKTFGA
LSATHRAAYQDVFFAPAEGFDKVPYGDVAALEQALAARPGEYAAFLVEPIQGEGGIVVPPPGYLRAAREICRRHGVLFIVD
EVQTGLGRTGALFACQAEGVTPDAMTLAKALGGGLMPIGAVLCTEEVYTEEFATKHSSTFAGNTLACRAGLAALDLLTRDD
QALVRHVAETGEYLRQGLLAIQRRHPRVIREVRGRGFMLGLQFGVTRETFPGNLLGVMGEQELLTPVIASYLLNVEGLRVA
PTLNGADVIRIEPPLIATREECDYALAAIERVVDLIDREDTAGLLRHLVGQGRVGQDRHGATSPDHGMNGRSAAIGRVVVG
RSGTGVTRDGDGASRRATGAGELPATGRGGQVGTGTVHGGASQTPAAGSSREEPATPSDDPAEGRFAFLVHPVDLENYPEF
DPGLAAFTREELADLAGRWNHLLKPFRIGQTRVVSTCGRTAYGEFYVVPRTADELLAMPAQEAVAAVKEALDLARDGGARI
VGLGAYTSVVTRGGLHLRDAGVALTTGNSFTVAAAVEAIDEATRRLGFPLAESTVAVVGATGAIGRATALLLGPRVQRLLL
IGNPARPEQSRRRLLRVAGEICRHVLVSAGRPSPVGRAGRVDDGDRDQENHGAPLGGSGAAFFNRFGTAEAGELAPLAAQL
LDFGGWPNADAPVEEFVRRLETWMARGQCPLVITTDLDAMLPLADVVVTATSSTAHLVTPGNVKFGAVVCDLSRPPNVSRE
VRDARPDVLVIDGGVIEVPGRPSLGWNFGFERGLVYACMAETMILALEHHYQHTSLGADLNLETILWLEELARKHGFRLAQ
LRSFDRPLPDEAWERLVAARSAVLGSAAR

Fig. S17. *Tm*Fusion protein sequence. The protein sequence of *Tm*Fusion with a N-terminal His_6-tag. The start methionine (M) residue is shown in bold. The N-terminal His_6-tag is highlighted in green; the thrombin peptide sequence is highlighted in turquoise.

2.2.2 *Tm*TAm protein sequence

MGSSHHHHHHSSGLVPRGSH**M**HPFRQYVNPHLGELLEQIQMDKRFVRGQGCWLWDEDGRRYLDFVAAYGALPFGFNPPEIW
AALEEARRQGEPSFIQPSFLNAAGELARRLIEVAPPGLRYVTFANSGAEAVEAAIKLARAATGRPRILSTENAFHGKTFGA
LSATHRAAYQDVFFAPAEGFDKVPYGDVAALEQALAARPGEYAAFLVEPIQGEGGIVVPPPGYLRAAREICRRHGVLFIVD
EVQTGLGRTGALFACQAEGVTPDAMTLAKALGGGLMPIGAVLCTEEVYTEEFATKHSSTFAGNTLACRAGLAALDLLTRDD
QALVRHVAETGEYLRQGLLAIQRRHPRVIREVRGRGFMLGLQFGVTRETFPGNLLGVMGEQELLTPVIASYLLNVEGLRVA
PTLNGADVIRIEPPLIATREECDYALAAIERVVDLIDREDTAGLLRHLVG

Fig. S18. *Tm*TAm protein sequence. The protein sequence of the *Tm*Fusion N-terminal domain (1-435 aa) (*Tm*TAm) with a N-terminal His_6-tag. The start methionine (M) residue is shown in bold. The N-terminal His_6-tag is highlighted in green; the thrombin peptide sequence is highlighted in turquoise.

2.2.3 *Tm*DHR protein sequence

MGSSHHHHHHSSGLVPRGSH**M**QGRVGQDRHGATSPDHGMNGRSAAIGRVVVGRSGTGVTRDGDGASRRATGAGELPATGRG
GQVGTGTVHGGASQTPAAGSSREEPATPSDDPAEGRFAFLVHPVDLENYPEFDPGLAAFTREELADLAGRWNHLLKPFRIG
QTRVVSTCGRTAYGEFYVVPRTADELLAMPAQEAVAAVKEALDLARDGGARIVGLGAYTSVVTRGGLHLRDAGVALTTGNS
FTVAAAVEAIDEATRRLGFPLAESTVAVVGATGAIGRATALLLGPRVQRLLLIGNPARPEQSRRRLLRVAGEICRHVLVSA
GRPSPVGRAGRVDDGDRDQENHGAPLGGSGAAFFNRFGTAEAGELAPLAAQLLDFGGWPNADAPVEEFVRRLETWMARGQC
PLVITTDLDAMLPLADVVVTATSSTAHLVTPGNVKFGAVVCDLSRPPNVSREVRDARPDVLVIDGGVIEVPGRPSLGWNFG
FERGLVYACMAETMILALEHHYQHTSLGADLNLETILWLEELARKHGFRLAQLRSFDRPLPDEAWERLVAARSAVLGSAAR

Fig. S19. *Tm*DHR protein sequence. The protein sequence of the *Tm*Fusion C-terminal domain (436-981 aa) (*Tm*DHR) with a N-terminal His_6-tag. The start methionine (M) residue is shown in bold. The N-terminal His_6-tag is highlighted in green; the thrombin peptide sequence is highlighted in turquoise.

2.3 Thermaerobacter subterraneus DSM 13965 ThesuDRAFT_00745

2.3.1 *Ts*Fusion protein sequence

MGSSHHHHHHSSGLVPRGSH**M**HPFRQYVNPHLAELLEQIQMDKRFVRGEGCWLWDDQGRRYLDFVAAYGALPFGFNPPEIW
AALEEARHRGEPSFVQPSFLNAAGELARRLIEVAPPGLRYVTFANSGAEAVEAAIKLARAATGRPRILSTDNGFHGKTFGA
LSATHRAAYQDAFFAPAEGFDKVPYGDLEALERALAARPGEYAAFLVEPIQGEGGIVVPPPGYLREAREVCRRHGVLFIAD
EVQTGLGRTGVLFACQAEGVTPDAITLAKALGGGLMPIGAVLCTEEVYTEEFATKHSSTFAGNTLACRAGLAALDLLTRDE
QALVRHVAETGEYLRQGLLAIQRRHPRVIREVRGRGFMLGIQFGVDREAFPGNLLGVMGEQELLTPVIASYLLNVEGLRVA
PTLNGADVIRIEPPLIATRAECDYALAAIERVVELIDRDDTAALLRHLVSPQATTGQAVDRPARHQPALLAVRSREPEEAV
APSGDPREGRFAFLVHPVDLENYPEFDPSLAAFSRAELEELAGRWNHLLKPFRIGRTRVVSAAGATAYGEFYAVPRTADEF
LAMPQAEAVAEIEEAIELARENGARIVGLGAYTSVVTRGGLHLRDAGVALTTGNSFTVVAAVEAIAEASRRLGFPLGQGTV
AVVGATGAIGRATALLLAGEVRRLVLIGNPARPEQSRRRLLRVAADLARHVLSLAASGRPLGPLAQVLVEFGGWPDPAEPA
EAFLPRLEAWLAAGRCPLVVTTDLDAMLPQADVVVTATSSTAHLVTPRNVKFGAVVCDLSRPPNVSREVGDARPDVLVIDG
GVIEVPGRPSMGWNFGFERGLVYACMAETMMLALEHHYRHTSLGADLNLETILWLKDLARQHGFRLAELRSFDRPLPAEAW
ERVLAARSALGTAAR

Fig. S20. *Ts*Fusion protein sequence. The protein sequence of *Ts*Fusion with a N-terminal His_6-tag. The start methionine (M) residue is shown in bold. The N-terminal His_6-tag is highlighted in green; the thrombin peptide sequence is highlighted in turquoise.

2.3.2 *Ts*TAm protein sequence

MGSSHHHHHHSSGLVPRGSH**M**HPFRQYVNPHLAELLEQIQMDKRFVRGEGCWLWDDQGRRYLDFVAAYGALPFGFNPPEIW
AALEEARHRGEPSFVQPSFLNAAGELARRLIEVAPPGLRYVTFANSGAEAVEAAIKLARAATGRPRILSTDNGFHGKTFGA
LSATHRAAYQDAFFAPAEGFDKVPYGDLEALERALAARPGEYAAFLVEPIQGEGGIVVPPPGYLREAREVCRRHGVLFIAD
EVQTGLGRTGVLFACQAEGVTPDAITLAKALGGGLMPIGAVLCTEEVYTEEFATKHSSTFAGNTLACRAGLAALDLLTRDE
QALVRHVAETGEYLRQGLLAIQRRHPRVIREVRGRGFMLGIQFGVDREAFPGNLLGVMGEQELLTPVIASYLLNVEGLRVA
PTLNGADVIRIEPPLIATRAECDYALAAIERVVELIDRDDTAALLRHLVS

Fig. S21. *Ts*TAm protein sequence. The protein sequence of the *Ts*Fusion N-terminal domain (1-435 aa) (*Ts*TAm) with a N-terminal His_6-tag. The start methionine (M) residue is shown in bold. The N-terminal His_6-tag is highlighted in green; the thrombin peptide sequence is highlighted in turquoise.

2.3.3 *Ts*DHR protein sequence

MGSSHHHHHHSSGLVPRGSH**M**PQATTGQAVDRPARHQPALLAVRSREPEEAVAPSGDPREGRFAFLVHPVDLENYPEFDPS
LAAFSRAELEELAGRWNHLLKPFRIGRTRVVSAAGATAYGEFYAVPRTADEFLAMPQAEAVAEIEEAIELARENGARIVGL
GAYTSVVTRGGLHLRDAGVALTTGNSFTVVAAVEAIAEASRRLGFPLGQGTVAVVGATGAIGRATALLLAGEVRRLVLIGN
PARPEQSRRRLLRVAADLARHVLSLAASGRPLGPLAQVLVEFGGWPDPAEPAEAFLPRLEAWLAAGRCPLVVTTDLDAMLP
QADVVVTATSSTAHLVTPRNVKFGAVVCDLSRPPNVSREVGDARPDVLVIDGGVIEVPGRPSMGWNFGFERGLVYACMAET
MMLALEHHYRHTSLGADLNLETILWLKDLARQHGFRLAELRSFDRPLPAEAWERVLAARSALGTAAR

Fig. S22. *Ts*DHR protein sequence. The protein sequence of the *Ts*Fusion C-terminal domain (436-886 aa) (*Ts*DHR) with a N-terminal His_6-tag. The start methionine (M) residue is shown in bold. The N-terminal His_6-tag is highlighted in green; the thrombin peptide sequence is highlighted in turquoise.

2.4 Thermincola ferriacetica Z-0001 Tfer_2018

2.4.1 *Tf*Fusion protein sequence

MGSSHHHHHHSSGLVPRGSH**M**KFSSLNPTMDKLFECFKLDVAYVKGEGTYLYDQAGNKYLDFIAQYGAVPFGYNPPELVAA
AKKYFDLSLPSMVQPSIPVKAVELAEMLLQLAPGEMAQATFCQSGAEAVEVAIKLARSTTGKPKILSTKNSFHGKTMGALS
ATGRDVYQKPFFTPVPGFEHIPFGDLDMLENKMRTEGKQIAAFLVEPIQGEGGIIVPPEGYLKNAEIICRKYGVLLAVDEI
QTGLGRTGELFACDREGVEPDILLLSKALGGGLVPLGVCLSTRQAWNEEFGRLHSSTFANNNFTCAIGLAVLNKLLENGRQ
LINNAKSAGNYLLKNLEDINRQYPGVIKEIRGRGLMLGLEFNEFDGSESFSMKYLAEQGGFSPLLAGYLLNVHKVRVAPFL
NNPMTLRLQPSLTVATVEIDRALHGLERVVKALYYQDHCELYSYIIGKEPGPIRDFRSEKKAVTGSELLPEEKPTEKFAFL
IHYPSSEDVIKNNPSFKKASKDILEKLIDWEASLDAEPEVIVHLPAIKSKAGKIAEGWLIGIPYSGRHMMEMPRKDAVKVL
VTALDKAKALGARIVGLGAYTSVVSRGGSDLQGKGIAVTSGNSYTIATAFDALIEGARLMGIDPAESTGCVIGATGSIGRV
CAILLAEEIDKLVLVGNPEKEKTSLRRMEQLADEIYTRAFREILAYKGKVAKIKGIAKWLKGFLDRKRKDDPEVWNKLLTA
LENQGFSINGYIHENLAAKGKYSNPPVKITVNIKQALLQSDLIISASNSTNHLIGPGHLKPGSVICDVARPPDVSEAVLEQ
RKDVLVIEGGLVQYPDDICFGQNMGYEPGVNMACLSETMLLALEGTYRDFSIGLKIPVENVYYLRELAQRHGFKLATPWNK
NGGVTPEVARMIKEAALKNENVEKIKTPKSS

Fig. S23. *Tf*Fusion protein sequence. The protein sequence of *Tf*Fusion with a N-terminal His_6-tag. The start methionine (M) residue is shown in bold. The N-terminal His_6-tag is highlighted in green; the thrombin peptide sequence is highlighted in turquoise.

2.4.2 *Tf*TAm protein sequence

MGSSHHHHHHSSGLVPRGSH**M**KFSSLNPTMDKLFECFKLDVAYVKGEGTYLYDQAGNKYLDFIAQYGAVPFGYNPPELVAA
AKKYFDLSLPSMVQPSIPVKAVELAEMLLQLAPGEMAQATFCQSGAEAVEVAIKLARSTTGKPKILSTKNSFHGKTMGALS
ATGRDVYQKPFFTPVPGFEHIPFGDLDMLENKMRTEGKQIAAFLVEPIQGEGGIIVPPEGYLKNAEIICRKYGVLLAVDEI
QTGLGRTGELFACDREGVEPDILLLSKALGGGLVPLGVCLSTRQAWNEEFGRLHSSTFANNNFTCAIGLAVLNKLLENGRQ
LINNAKSAGNYLLKNLEDINRQYPGVIKEIRGRGLMLGLEFNEFDGSESFSMKYLAEQGGFSPLLAGYLLNVHKVRVAPFL
NNPMTLRLQPSLTVATVEIDRALHGLERVVKALYYQDHCELYSYIIG

Fig. S24. *Tf*TAm protein sequence. The protein sequence of the *Tf*Fusion N-terminal domain (1-432 aa) (*Tf*TAm) with a N-terminal His_6-tag. The start methionine (M) residue is shown in bold. The N-terminal His_6-tag is highlighted in green; the thrombin peptide sequence is highlighted in turquoise.

2.4.3 *Tf*DHR protein sequence

MGSSHHHHHHSSGLVPRGSH**M**KEPGPIRDFRSEKKAVTGSELLPEEKPTEKFAFLIHYPSSEDVIKNNPSFKKASKDILEK
LIDWEASLDAEPEVIVHLPAIKSKAGKIAEGWLIGIPYSGRHMMEMPRKDAVKVLVTALDKAKALGARIVGLGAYTSVVSR
GGSDLQGKGIAVTSGNSYTIATAFDALIEGARLMGIDPAESTGCVIGATGSIGRVCAILLAEEIDKLVLVGNPEKEKTSLR
RMEQLADEIYTRAFREILAYKGKVAKIKGIAKWLKGFLDRKRKDDPEVWNKLLTALENQGFSINGYIHENLAAKGKYSNPP
VKITVNIKQALLQSDLIISASNSTNHLIGPGHLKPGSVICDVARPPDVSEAVLEQRKDVLVIEGGLVQYPDDICFGQNMGY
EPGVNMACLSETMLLALEGTYRDFSIGLKIPVENVYYLRELAQRHGFKLATPWNKNGGVTPEVARMIKEAALKNENVEKIK
TPKSS

Fig. S25. *Tf*DHR protein sequence. The protein sequence of the *Tf*Fusion C-terminal domain (433-902 aa) (*Tf*DHR) with a N-terminal His_6-tag. The start methionine (M) residue is shown in bold. The N-terminal His_6-tag is highlighted in green; the thrombin peptide sequence is highlighted in turquoise.

3. References

1 S. Sur, T. D. Romo and A. Grossfield, *J. Phys. Chem. B*, 2018, **122**, 2219–2226.

2 N. Stankovic, L. Senerovic, T. Ilic-Tomic, B. Vasiljevic and J. Nikodinovic-Runic, *Appl. Microbiol. Biotechnol.*, 2014, **98**, 3841–3858.

3 J. Franke, K. Ishida and C. Hertweck, *Angew. Chemie - Int. Ed.*, 2012, **51**, 11611–11615.

4 N. Rojas Murcia, X. Lee, P. Waridel, A. Maspoli, H. J. Imker, T. Chai, C. T. Walsh and C. Reimmann, *Front. Microbiol.*, 2015, **6**, 170.

5 X. Lee, Á. Fox, J. Sufrin, H. Henry, P. Majcherczyk, D. Haas and C. Reimmann, *J. Bacteriol.*, 2010, **192**, 4251–4255.

6 P. Messner, K. Steiner, K. Zarschler and C. Schäffer, *Carbohydr. Res.*, 2008, **343**, 1934–1951.

7 S. Matthies, P. Stallforth and P. H. Seeberger, *J. Am. Chem. Soc.*, 2015, **137**, 2848–2851.

8 A. González, M. Rodríguez, A. F. Braña, C. Méndez, J. A. Salas and C. Olano, *Microb. Cell Fact.*, 2016, **15**, 56.

9 F. Kudo, Y. Kasama, T. Hirayama and T. Eguchi, *J. Antibiot. (Tokyo).*, 2007, **60**, 492–503.

10 J. A. Capra and M. Singh, *Bioinformatics*, 2007, **23**, 1875–82.

11 S. Lutz, *Curr. Opin. Biotechnol.*, 2010, **21**, 734–43.

12 L. A. Kelley, S. Mezulis, C. M. Yates, M. N. Wass and M. J. E. Sternberg, *Nat. Protoc.*, 2015, **10**, 845–858.

13 J. Jumper, R. Evans, A. Pritzel, T. Green, M. Figurnov, O. Ronneberger, K. Tunyasuvunakool, R. Bates, A. Žídek, A. Potapenko, A. Bridgland, C. Meyer, S. A. A. Kohl, A. J. Ballard, A. Cowie, B. Romera-Paredes, S. Nikolov, R. Jain, J. Adler, T. Back, S. Petersen, D. Reiman, E. Clancy, M. Zielinski, M. Steinegger, M. Pacholska, T. Berghammer, S. Bodenstein, D. Silver, O. Vinyals, A. W. Senior, K. Kavukcuoglu, P. Kohli and D. Hassabis, *Nature*, 2021, **596**, 583–589.

14 M. Varadi, S. Anyango, M. Deshpande, S. Nair, C. Natassia, G. Yordanova, D. Yuan, O. Stroe, G. Wood, A. Laydon, A. Žídek, T. Green, K. Tunyasuvunakool, S. Petersen, J. Jumper, E. Clancy, R. Green, A. Vora, M. Lutfi, M. Figurnov, A. Cowie, N. Hobbs, P. Kohli, G. Kleywegt, E. Birney, D. Hassabis and S. Velankar, *Nucleic Acids Res.*, 2022, **50**, D439–D444.

15 A. Rath, M. Glibowicka, V. G. Nadeau, G. Chen and C. M. Deber, *Proc. Natl. Acad. Sci. U. S. A.*, 2009, **106**, 1760–1765.

16 S. Prasad, P. B. Khadatare and I. Roy, *Appl. Environ. Microbiol.*, 2011, **77**, 4603–4609.
